# Genome-wide association studies for a comprehensive understanding of the genetic architecture of culm strength and yield traits in rice

**DOI:** 10.3389/fpls.2023.1298083

**Published:** 2024-01-22

**Authors:** Jyothi Badri, Revadi Padmashree, Chandrappa Anilkumar, Akshay Mamidi, Subhakara Rao Isetty, AVSR Swamy, Raman Menakshi Sundaram

**Affiliations:** ^1^ Crop Improvement Section, ICAR-Indian Institute of Rice Research (ICAR-IIRR), Hyderabad, India; ^2^ Crop Improvement Section, ICAR-National Rice Research Institute (ICAR-NRRI), Cuttack, India; ^3^ Department of Genetics and Plant Breeding, College of Agriculture, Professor Jayashankar Telangana State Agricultural University (PJTSAU), Hyderabad, India

**Keywords:** genotyping by sequencing (GBS), association mapping, lodging resistance, culm strength, marker trait associations (MTAs), candidate gene, Epistatic interactions

## Abstract

Lodging resistance in rice is a complex trait determined by culm morphological and culm physical strength traits, and these traits are a major determinant of yield. We made a detailed analysis of various component traits with the aim of deriving optimized parameters for measuring culm strength. Genotyping by sequencing (GBS)-based genome-wide association study (GWAS) was employed among 181 genotypes for dissecting the genetic control of culm strength traits. The VanRaden kinship algorithm using 6,822 filtered single-nucleotide polymorphisms (SNPs) revealed the presence of two sub-groups within the association panel with kinship values concentrated at<0.5 level, indicating greater diversity among the genotypes. A wide range of phenotypic variation and high heritability for culm strength and yield traits were observed over two seasons, as reflected in best linear unbiased prediction (BLUP) estimates. The multi-locus model for GWAS resulted in the identification of 15 highly significant associations (*p*< 0.0001) for culm strength traits. Two novel major effect marker–trait associations (MTAs) for section modulus and bending stress were identified on chromosomes 2 and 12 with a phenotypic variance of 21.87% and 10.14%, respectively. Other MTAs were also noted in the vicinity of previously reported putative candidate genes for lodging resistance, providing an opportunity for further research on the biochemical basis of culm strength. The quantitative trait locus (QTL) hotspot identified on chromosome 12 with the synergistic association for culm strength trait (section modulus, bending stress, and internode breaking weight) and grain number can be considered a novel genomic region that can serve a dual purpose of enhancing culm strength and grain yield. Elite donors in the *indica* background with beneficial alleles of the identified major QTLs could be a valuable resource with greater significance in practical plant breeding programs focusing on improving lodging resistance in rice.

## 1 Introduction

Lodging is a major constraint in rice, affecting both grain yield and quality, particularly in coastal areas prone to cyclonic weather. Lodged plants exhibit limited photosynthetic ability and dry matter production, as the stooping canopy causes losses in crop yield (Islam et al., 2007; Kashiwagi et al., 2010; Mariani and Ferrante, 2017). In severe cases, lodging can result in more than 50% yield loss or complete loss of the crop (Setter et al., 1997; Lang et al., 2012; Luo et al., 2022). It can also lead to pre-harvest sprouting of the panicles due to the high moisture content of the lodged plants. Lodging also poses difficulties in harvest operations, increases the cost of grain drying, and leads to rice mycotoxin contamination (Kashiwagi et al., 2005; Nakajima et al., 2008). The rice crop is prone to lodging from the mid-grain filling stage to the grain ripening, hardening, and maturity stages. The continuous translocation of organic matter to the spikelets from the culm sheath weakens the mechanical strength of the culm with an increase in mass in the top portions of the plant, i.e., increasing grain size grain filling, and grain weight, increasing the risk of lodging or making the culm vulnerable to lodging (Ishimaru et al., 2008).

A breakthrough “Green Revolution” in rice was successfully achieved with the introduction of high-fertilizer-responsive semi-dwarf varieties. In addition to doubling rice grain yield, the short stature conferred by the semidwarf1 (*sd1*) gene increased lodging resistance, making rice capable of supporting heavier panicles. Breeders were convinced of the suitability of semi-dwarf varieties for increased crop productivity, and many improved semi-dwarf varieties have been developed and cultivated worldwide (Peng et al., 2000; Ashikari et al., 2002; Asano et al., 2007; Hirano et al., 2017). The *Sd1* gene, which encodes the gibberellin synthesis enzyme, and the loss-of-function mutant *sd-1* allele reduce plant height and enhance lodging resistance (Sasaki et al., 2002). Thus, the semi-dwarf plant type has been for decades the main target in improving the lodging resistance and harvest index of rice (Keller et al., 1999; Peng and Khush, 2003). However, lodging remains a serious problem in widely adapted, high-yielding elite cultivars, despite the short stature conferred by the*sd1* gene. The susceptibility of rice plants to lodging varies even among cultivars with short plant height, but with more biomass (Terashima et al., 1992). Reduced culm strength in semi-dwarf varieties due to decreased culm diameter, thickness, and plant biomass has been reported (Okuno et al., 2014; Ookawa et al., 2016) as *sd1* is associated with short plant height; it also has negative pleiotropic effects on culm morphology in rice (Yano et al., 2015). Reducing the plant’s height also reduces its photosynthetic capacity and leads to a decrease in total biomass production, thus restricting the plant’s potential for further yield increases (Flintham et al., 1997; Murai et al., 2002; Okuno et al., 2014). These make it enigmatic to improve lodging resistance and, consequently, the harvest index by using the semi-dwarf trait alone. Further, attempts to increase the sink capacity, i.e., grain yield, will be successful only when the source efficiency is enhanced, which is the basis of the new plant type concept (Jyothi et al., 2018). Although an improved source–sink relationship is essential to maximize the harvest index, it also promotes biomass via increased stem and leaf elongation with an overall increase in plant height. Tall varieties are prone to lodging due to the breaking of the basal culm, while the plant type concept advocates semi-tall stature to achieve higher yields, thus making culm strength imperative to prevent discernible negative effects of lodging in high-yielding varieties.

Lodging resistance is known to be a complex trait influenced by many interacting agro-morphological, biochemical, and anatomical traits. Different techniques have been used to measure culm strength and lodging resistance in rice. The bending moment at breaking, which is a function of section modulus and bending stress, was used as a measure for the physical strength of the culm (Ookawa et al., 2010). Section modulus is directly influenced by the culm morphology (diameter and wall thickness), and bending stress is a function of culm cell wall components, such as cellulose and lignin content. Pushing resistance measured with a prostrate tester is the most common parameter used in assessing the culm strength (Terashima et al., 1992; Won et al., 1998; Berry et al., 2003; Kashiwagi and Ishimaru, 2004). However, because the tiller number influences the pushing resistance of the lower part of the culm, bending stress was measured considering both the tiller number and prostrate tester readings ranging from 0 to 40 (Hai et al., 2005; Yadav et al., 2017; Jyothi et al., 2018). Resistance to prostrating on a visually rated scale of 0 (prostrating) to 1 (no or little prostrating) was also used for measuring lodging resistance to typhoons (Kashiwagi and Ishimaru, 2004; Zhao et al., 2023). Recently, gravity center height, along with the breaking strength of the basal internode and culm morphology traits, was used in the measurement of lodging resistance traits (Yang et al., 2023). It is now evident that the culm strength parameter is described in different ways by researchers, and efforts are leading toward the development of an optimized parameter that truly reflects the strength of the culm.

To understand the physiological and morphological basis of culm strength and improve it further, prior knowledge of the genetic control of mechanisms that regulate culm strength is a prerequisite. Identification of genes can hasten precision breeding aimed at improving lodging resistance. A number of quantitative trait locus (QTL) mapping studies for culm length, culm strength, and culm thickness related to lodging resistance have been carried out using different segregating populations in rice (Kashiwagi and Ishimaru, 2004; Mu et al., 2004; Kashiwagi et al., 2008; Zhu et al., 2008; Ookawa et al., 2010; Yano et al., 2015; Yadav et al., 2017; Nomura et al., 2019; Yang et al., 2023; Zhao et al., 2023). To elucidate the molecular mechanisms of complex traits like lodging resistance and associated culm-related traits, a genome-wide association study (GWAS) is a promising strategy to exploit the abundant genetic diversity of rice captured among the diverse rice germplasm. GWAS uses numerous historic recombinations in a large natural population and offers the potential to localize the trait genetic determinants effectively to a narrower region (Bollinedi et al., 2020). Further, the application of single-nucleotide polymorphism (SNP) in a GWAS study provides dense coverage of markers in the entire genome, which helps to identify the functional variation governing the trait in a more precise manner. The results of GWAS can be used either for dissecting mechanisms of lodging resistance or for improving the prediction accuracy of genome-wide predictions in genomic selection programs (Anilkumar et al., 2023).

In this milieu, we hypothesized that the diverse germplasm panel tested over two seasons would have the potential to reveal the candidate genes that regulate culm strength and lodging resistance. We tested the hypothesis by adapting the GWAS approach with a multi-locus model for detecting genomic regions associated with the target traits. The present investigation aimed to identify (1) the genomic regions associated with culm strength, (2) the genetic associations between culm strength and yield traits, (3) the putative candidate genes within the identified genetic regions, and (4) a useful genetic resource for concurrently boosting culm strength and lodging resistance and provide a basis for further research on lodging resistance mechanisms in rice.

## 2 Materials and methods

### 2.1 Plant material and field experiments

**Figure 1:**
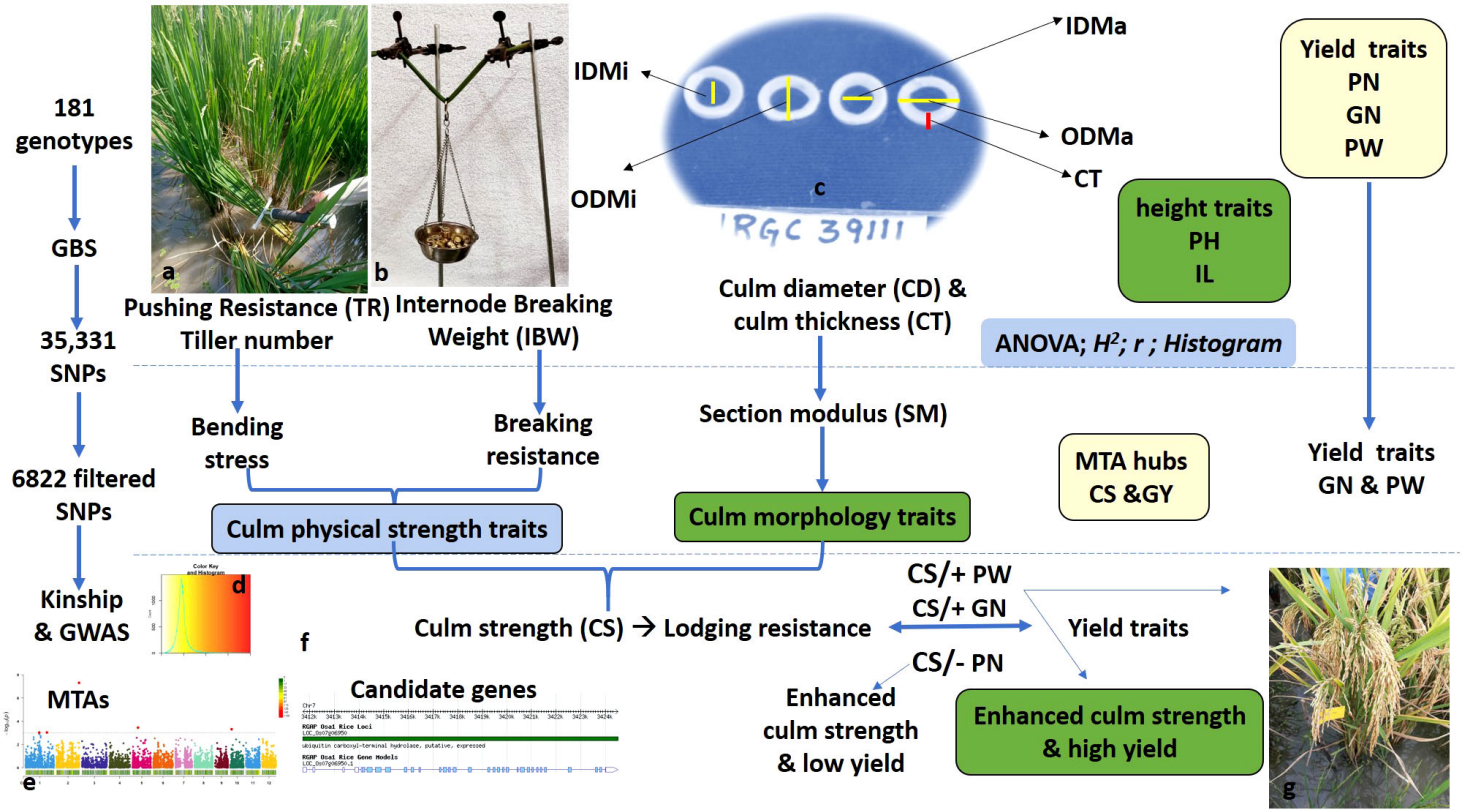
Schematic flow of the experimental strategy, including phenotypic evaluation, trait grouping for culm strength and yield traits, genome-wide studies for marker–trait associations, and identification of putative candidate genes. **(A)** Measurement of pushing resistance using a prostrate tester in the field. TR, tester reading with prostrate testing as a measure of pushing resistance. **(B)** Measurement of internode breaking weight in increments of 25 g on culm samples for basal internodes. **(C)** Measurement of culm morphology traits using digital Vernier calipers. ODMa, outer diameter in mm on the major axis; ODMi, outer diameter in mm on the minor axis; IDMa, inner diameter in mm on the major axis; IDMi, inner diameter in mm on the minor axis; CD, culm diameter in mm; CT, culm wall thickness in mm; PH, plant height in cm; IL, basal internode length in cm. **(D)** Color key and histogram depicting population structure. **(E)** A representative Manhattan plot for genomic regions associated with culm strength traits. **(F)** A representative candidate gene within the identified QTL region. GBS, genotyping by sequencing; MTAs, marker–trait associations. **(G)** A representative plant with a high yield (high grain number) and strong culm. PN, panicle number; GN, grain number; PW, panicle weight in g; GY, grain yield in g; CS, culm strength; QTL, quantitative trait locus.

The study consisted of an association mapping panel comprising 181 diverse genotypes, which included tropical *japonica* accessions (TrJ: 30), *indica* landraces (Ind-L: 7), *indica* cultivars (Ind-C: 26), *indica*/*indica*-derived lines (Ind-D: 55), and *indica*/tropical *japonica*-derived lines (ITrJ-D: 63). TrJ and Ind-L are from a global collection maintained at ICAR-IIRR, Hyderabad, India; Ind-C are released cultivars collected from all across the country; Ind-D are multiparent-derived, marker-assisted forward breeding introgression lines in diverse elite backgrounds; and ITrJ-D are from the crosses of multiparent elite *indica*/tropical *japonica* genotypes developed at ICAR-IIRR (**Supplementary Table S1**). The genetic purity of experimental material was maintained by continual self-pollination over the years. The complete set of genotypes was evaluated in two cropping seasons at ICAR-IIRR, Rajendranagar farm (17°19′N; 78°23′E, 542 m) during the dry seasons (DS) of 2022 and 2023. An augmented randomized complete block design (ARCBD) was adopted for field evaluation in both years. For the experimental design, 10 genotypes, including one TRJ accession (IRGC 39111), five released cultivars (i.e., DRR Dhan 54, DRR Dhan 48, Improved Samba Mahsuri, Samba Mahsuri, Swarna), and four Y-BILs (i.e., RMS 2077, RMS 2097, RMS 2495, and RMS 2509) were used as checks. The experiment was laid out in four blocks, replicating the checks block-wise and randomizing the test treatments across all blocks. The seeds were germinated on a raised seedbed to ensure uniform germination and 28-day-old seedlings were transplanted into a puddled rice field. In DS 2022, the experimental material was sown on 6 January 2022, and transplanted on 5 February 2022. In DS 2023, the experimental material was sown and transplanted on 26 December 2022, and 22 January 2023, respectively. Each genotype was planted in two rows at 14 hills per row with a 20-cm spacing between rows and a 15-cm spacing between plants. The recommended package of practices was adopted to ensure a good crop stand in both seasons. The lines were subjected to phenotypic evaluation of culm strength and yield-associated traits and genomic studies for the discovery of marker–trait associations and identification of putative candidate genes (schematically illustrated in **Figure 1**).

### 2.2 Evaluation of morphological traits

A total of 12 quantitative traits were evaluated, *viz*., plant height, length of the basal internode, culm thickness, culm diameter, pushing resistance as a measure of prostrate tester reading, tiller number, bending stress, section modulus, breaking resistance as a measure of internode breaking weight, panicle number, grain number, and panicle weight. For culm parameters, two plants were uprooted 20 days after heading, and data were collected using destructive sampling in the lab as described below. For yield parameters, while data were collected at maturity in the field on plant height, tiller number, and panicle number, two panicles were collected randomly at harvest for post-harvest data. Plants on the edges were avoided, and plants from the middle of the rows were chosen for data collection to avoid the border effect. Plant height (in cm) was measured as the length between the plant base and the panicle tip, and tiller number was measured as the total number of tillers, panicle number as the number of panicle-bearing tillers, grain number as the total number of filled grains in a panicle, and panicle weight as the weight of a fully matured panicle.

#### 2.2.1 Measurement of culm morphology traits

Destructive sampling was used for the measurement of culm traits 20 days after heading. Two plants were uprooted from the main plot, and the lower internode (second basal internode) of the main culms was sampled for recording the observations. The second basal internodes were dissected transversely at the mid-point, and the culm diameter was measured using digital Vernier calipers.

Culm thickness (in mm) was measured as the average difference between the outer and inner diameters on the major and minor axes.


$$\text{Culm thickness }\left(\text{CT}\right)=\left[\frac{\left(a_{1}+b_{1}\right)}{2}\right]-\left[\frac{\left(a_{2}+b_{2}\right)}{2}\right].$$

Culm diameter (in mm) was measured as the average of the culm outer diameter on the minor and major axes using digital Vernier calipers (**Figure 1C**).


$$\text{Culm diameter }\left(\text{CD}\right)=\;\frac{\left(a_{1}+b_{1}\right)}{2}.$$

Section modulus was determined as described by Ookawa et al. (2010); Ookawa and Ishihara (1992) using the following formula:


Section modulus(SM)=π32×(a1  3b1−a2  3b2)a1,

where a_1_ is the outer diameter of the minor axis in an oval cross-section, b_1_ is the outer diameter of the major axis in an oval cross-section, a_2_ is the inner diameter of the minor axis in an ovalcross-section, and b_2_ is the inner diameter of the major axis in an oval cross-section.

#### 2.2.2 Measurement of culm physical strength traits

The prostrate tester (DIK-7400, Daiki Rika Kogyo Co. Ltd., Tokyo, Japan) was used to measure the pushing resistance of the culm (**Figure 1A**), and bending stress was calculated as described by Hai et al. (2005) using the following formula:


$$\text{Bending stress }\left(\text{BS}\right)=\left(\frac{TR}{40}\right)\times \left(\frac{1,000}{TN}\right),$$

where TR is the prostrate tester reading value (a measure of pushing resistance) and TN is the tiller number.

Internode breaking weight (IBW) in increments of 25 g was used as a measure of breaking resistance. IBW was analyzed using a supporting structure composed of two burette stands and clumps spaced at the length of each internode analyzed (**Figure 1B**). The internode was arranged horizontally with the portions of the terminal nodes held on the clamps of each burette stand, with the addition of an empty container (previously weighed) hanging in the central part of the internode. Weights of the determined mass (25 g) were cumulatively added to the container at regular intervals of 1 second until the internode flexed and collapsed.

### 2.3 Genotyping by sequencing and marker data generation

Fresh leaf tissue samples from four to six plants per line were collected 20 days after transplanting. The next-generation sequencing (NGS) platform Illumina NovaSeq was used for genotyping by sequencing (GBS). Raw sequence data were processed to obtain high-quality clean reads using Trimmomatic, v. 0.38. The reads of the samples were aligned to the *Oryza sativa indica* reference genome, https://plants.ensembl.org/Oryza_indica/Info/Annotation/#assembly using BWA MEM (v. 0.7.17), with minimum seed length set to 32 and shorter split hits marked as secondary (parameters: -k 32 –M). The mpileup utility of Samtools (v. 0.1.18) was used to identify SNPs from the sorted BAM file of the sample. The SNPs were filtered based on a minimum read depth of 5 and a quality threshold of 25. Markers without clear physical position information on the chromosomes were removed. The genotyping data were filtered by removing markers with missing values >20% and minor allele frequency (MAF)<5%.

### 2.4 Genome-wide association analysis

The average measurements of all the traits recorded in each season were subjected to the estimation of best linear unbiased predictions (BLUP) that were used in further analysis. In the case of checks, the data of all the replications were averaged and used in BLUP estimates. Average values in each year were used to estimate, across years, the BLUP values for each trait. The across-year BLUP values for all the traits measured on experimental genotypes and SNP marker information were used as inputs for GWAS analysis. Genome-wide association analysis was performed following a multi-locus analysis method using the genome association and prediction integrated tool (GAPIT) v.3 software package in R (Lipka et al., 2012). The relatedness between individuals in the population was delineated by estimating a kinship similarity matrix (K) and population structure (Q) modeled using principal component analysis (PCA) as random and fixed effects, respectively (Yu et al., 2006), making the model more stringent and able to control false-positive marker–trait associations (MTAs). The multi-locus-based GWAS model of BLINK (Huang et al., 2019) was applied for MTA analysis. Population structure and the kinship similarity matrix were also incorporated according to the model type. The BLINK model is associated with an efficient mixed model approach (EMMA), which corrects for population structure and simultaneously identifies the marker–trait associations. The exploratory threshold with false discovery rate-corrected *p* ≤ 0.001 (−log_10_ *p* ≥ 3) was used to report significant marker–trait associations.

### 2.5 Identification of putative candidate genes

After significant MTAs were identified using data from multiple environments, the flanking sequence spanning 150 kb upstream and downstream of the significant SNP position was used as a query against the Nipponbare rice reference genome, IRSGP1.0 RefSeq, v.1.1. Subsequently, JBrowse was used to examine candidate genes residing in chromosomal regions harboring significant MTA. The genes within the search limits were filtered based on the reported functions, and only those genes that are known to have an impact on plant development were shortlisted as putative candidate genes that regulate culm strength.

## 3 Results

### 3.1 Variation for culm strength- and yield-related traits in the association panel

**Figure 2:**
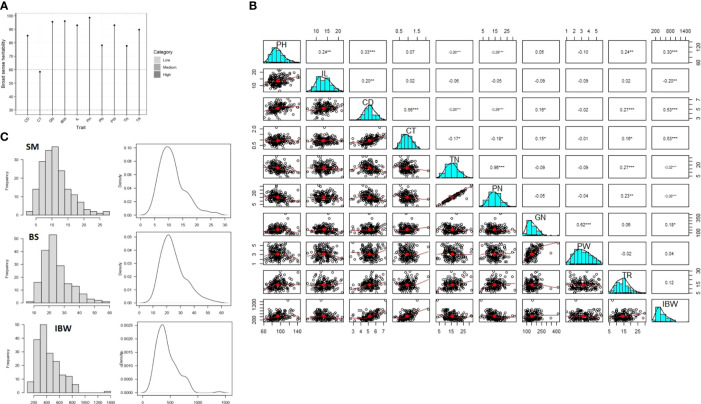
Genetic and phenotypic parameters for the components of culm strength and yield traits in the association panel. **(A)** Broad-sense heritability for seven culm strength (PH, plant height; IL, lower internode length; CD, culm diameter; CT, culm wall thickness; IBW, internode breaking weight [breaking resistance]; TR, prostrate tester reading value [pushing resistance]; TN, tiller number) traits and three yield (PN, panicle number; GN, grain number; PW, panicle weight) traits. **(B)** Correlation matrix showing correlation coefficients among seven culm strength traits and three yield traits. **(C)** Histograms showing the phenotypic distribution of section modulus (SM), bending stress (BS), and breaking resistance as a measure of internode breaking weight (IBW). *p < 0.05, **p < 0.01, ***p < 0.001.

Broad-sense heritability was high (79 to 99) for all the studied traits except for culm thickness, which had moderate heritability (59) (**Figure 2A**). BLUP values were calculated to further remove the environmental effects from trait data, and highly significant differences (*p*< 0.001) were found among the genotypes in the association panel for all the traits (**Table 1**). For example, it varied from 61.62 cm to 142.5 cm for plant height, 6.48 cm to 20.87 cm for lower internode length, 2.68 mm to 7.29 mm for culm diameter, 0.01 mm to 2.21 mm for culm thickness, 5.11 to 29.63 for pushing resistance as a measure of prostrate tester reading, 3.22 to 31.72 for tiller number, 116 g to 1,506 g for breaking resistance as a measure of internode breaking weight, 2.38 to 31.43 for panicle number, 79.82 to 410 for grain number, and 0.97 to 5.51 for panicle weight. Third-degree statistics-skewness and fourth-degree statistics-kurtosis were employed to understand the distribution of phenotypes in the population. The skewness of the population for all the traits was positively significant except for lower internode length, culm diameter, and panicle weight. However, kurtosis for all the traits except lower internode length and panicle weight was greater than three, indicating a leptokurtic distribution of phenotypes in the population (**Table 1**). The mean phenotypic data on culm strength and yield traits among the 181 genotypes in the association panel are provided in **Supplementary Table S2**.

**Table 1:** Phenotypic variation and distribution pattern of culm strength and grain yield traits in the association mapping panel used in the study.

Trait code	Trait name	Mean	SE	SD	Min	Max	Skewness	Kurtosis	MSS
PH	Plant height (cm)	93.42	1.11	14.92	61.62	142.50	0.77**	3.65^ns^	249**
IL	Lower internode length (cm)	13.53	0.21	2.88	6.48	20.87	0.35^ns^	2.56^ns^	9.36**
CD	Culm diameter (mm)	5.17	0.06	0.78	2.68	7.29	−0.10^ns^	3.86*	0.69**
CT	Culm thickness (mm)	0.93	0.02	0.31	0.01	2.21	0.51**	4.02*	0.08**
IBW	Internode breaking weight (g)	435	15.48	208.	115.64	1,506.47	1.24**	5.85**	47,398**
TR	Prostrate tester reading	14.47	0.33	4.48	5.11	29.63	0.59**	3.49^ns^	20.27**
TN	Tiller number	15.95	0.38	5.14	3.22	31.72	0.40*	3.15^ns^	27.05**
SM	Section modulus	11.18	0.41	5.46	1.25	30.22	1.04**	4.22**	30.88**
BS	Bending stress	24.84	0.73	9.84	5.95	59.76	0.93**	3.74ns	107.96**
PN	Panicle number	14.76	0.36	4.80	2.38	31.43	0.42*	3.51^ns^	23.51**
GN	Grain number	155	3.61	48.63	79.82	410	1.39**	7.11**	2,607**
PW	Panicle weight (g)	3.19	0.08	1.07	0.97	5.51	0.21^ns^	2.16**	1.24**

CV, coefficient of variation; SE, standard error; SD, standard error; Min, minimum; Max, maximum; MSS, mean sum of squares. *p < 0.05, **p < 0.01. ns, non-significant.

A correlation analysis among the seven culm strength traits and three yield traits was performed to understand the influence of variables on lodging resistance. A significant positive correlation was observed between culm morphology traits and breaking resistance (IBW), except for a negative correlation with internode length. Pushing resistance (TR) also correlated positively with culm morphology and breaking resistance, but it was not found to be statistically significant. Further, tiller number was negatively correlated with culm morphology traits and breaking resistance but positively correlated with pushing resistance (**Figure 2B**). These results suggest that culm morphology traits and breaking resistance have a direct influence on culm strength while pushing resistance is influenced by tiller number. Hence, both pushing resistance and tiller number should be considered in measuring the bending stress of the lower portion of the culm. Although breaking resistance showed a positive correlation with plant height, it showed a negative correlation with lower internode length, while pushing resistance showed a positive correlation with both height-related traits. Considering the indefinite relationship between culm strength and height traits in the present study, height traits were excluded from further analyses. While a positive correlation was observed for culm morphological and physical strength traits with grain number, correlations were negative with panicle number and had a non-significant association with panicle weight (**Figure 1B**). These findings suggest that the relationship of culm strength traits with grain number is synergistic, with panicle number being a tradeoff.

Based on the correlation analysis, the traits identified as key influencers of lodging resistance were: a) section modulus as a function of culm morphology traits; b) bending stress as a measure of pushing resistance and tiller number; and c) breaking resistance as a measure of internode breaking weight. The phenotypic distribution pattern in the form of histograms and density curves is depicted in **Figure 2C**. Section modulus varied from 1.64 mm^3^ to 30.6 mm^3^ with a mean of 11.18 mm^3^, bending stress ranged from 6.51 to 59.64 with a mean of 24.84, and internode breaking weight varied from 116 g to 1,506 g with a mean of 435 g. The normality of the trait distributions suggests that they may be controlled by several minor effect loci (**Figure 2C**). These three traits were further chosen for studying marker–trait associations.

### 3.2 Genome-wide distribution of SNPs

**Figure 3:**
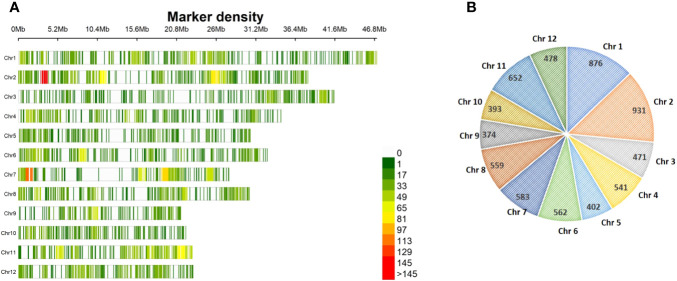
Genome-wide distribution of 6,822 SNPs across 12 chromosomes among 181 rice genotypes in the association panel. **(A)** Linkage map with 6,822 SNPs; the color of the vertical line indicates SNP density in a 1-Mb window. **(B)** Graphical representation of the chromosome-wide distribution of SNPs. SNPs, single-nucleotide polymorphisms.

A total of 3,72,087 sites obtained by GBS from 181 genotypes in the association mapping panel were subjected to further filtration in order to retain the sites with SNPs. A total site count of 35,331 was achieved after all the filtration. After removing the SNPs with MAF< 5%, the final 6,822 SNPs were used in the construction of a linkage map depicting the genome-wide distribution of SNPs on the 12 rice chromosomes (**Figure 3A**). The SNPs spanned a total genome size of 369.72 Mb, with the highest number of 931 SNPs covering 38.04 Mb on chromosome 2 and the lowest number of 374 SNPs spanning 21.2 Mb on chromosome 9. The maximum genome coverage of 46.86 Mb with 876 SNPs was on chromosome 1, followed by 41.10 Mb with 471 SNPs on chromosome 3 (**Figures 3A, B**).

### 3.3 Population structure and kinship analysis

**Figure 4:**
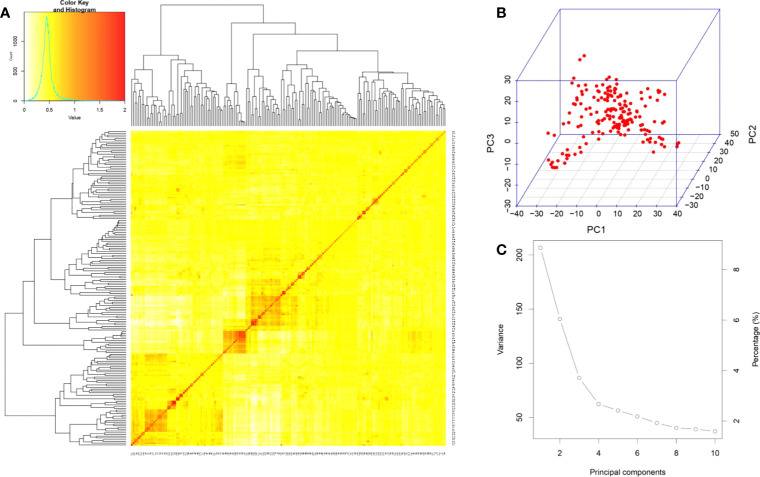
Population structure analysis of 181 genotypes in the association panel based on 6,822 SNPs. **(A)** Heatmap of the kinship matrix. The heatmap shows the level of relatedness among the population. The darker areas show a high level of relatedness between genotypes, and the dendrogram depicts the clustering of sub-populations. **(B)** 3D representation of principal component (PC) analysis showing no clear-cut grouping. **(C)** Scree plot depicting the number of significant PCs. There were three PCs that explained a cumulative variation of ∼22%, and nearly 10 PCs covered >95% of the variation in the population. SNPs, single-nucleotide polymorphisms.

The VanRaden kinship algorithm using the GAPIT was performed on the genome-wide 6,822 SNPs to ascertain the population structure and relatedness among genotypes in the association mapping panel. The relatedness in the association mapping (AM) panel was lower than the kinship index of 0.5, indicating less relatedness among the genotypes (**Figure 4A**). A lower level of relatedness among individuals in the GWAS panel reduces the false positives and increases the precision of the results. The kinship heatmap revealed the presence of two sub-groups within the AM panel and represented the distribution of genotypes within and between sub-populations (**Figure 4A**). There was no significant grouping of the genotypes, indicating an even distribution of the alleles in the panel, making it a perfect association mapping panel for GWAS. Principal component analysis provided insights into the SNP-based diversity in the association panel, which also detected the presence of two major population groups indicated by two significant components explaining the maximum variation in the association panel (**Figure 4B**). The scree plot (**Figure 4C**) showed that the first principal component (PC) explained the highest variation of 11%, followed by the second PC explaining 7%, and the third PC explaining 3.9% of the total variation. The variation explained by the first three PCs, which account for only 21.9% of the total variation, suggested the diverseness of the panel composition. The two major population groups in the panel were denoted as POP1 and POP2, and sub-populations within them as POP1A, POP1B, POP2A, and POP2B. It is interesting to note that there is no clear classification of *indica* and *japonica* in the sub-populations, and the genotypes belonging to both sub-species were interspersed in all the sub-population groups. POP2B was the largest and constituted 63.5% of the panel with 115 accessions, followed by POP1B, POP1A, and POP2A with 17.68, 11.60, and 7.18% of the panel, respectively. Sub-population-wise details of the genotypes of the AM panel in accordance with the kinship matrix are provided in **Supplementary Table S1**.

### 3.4 Genome-wide association studies for culm strength traits

**Figure 5:**
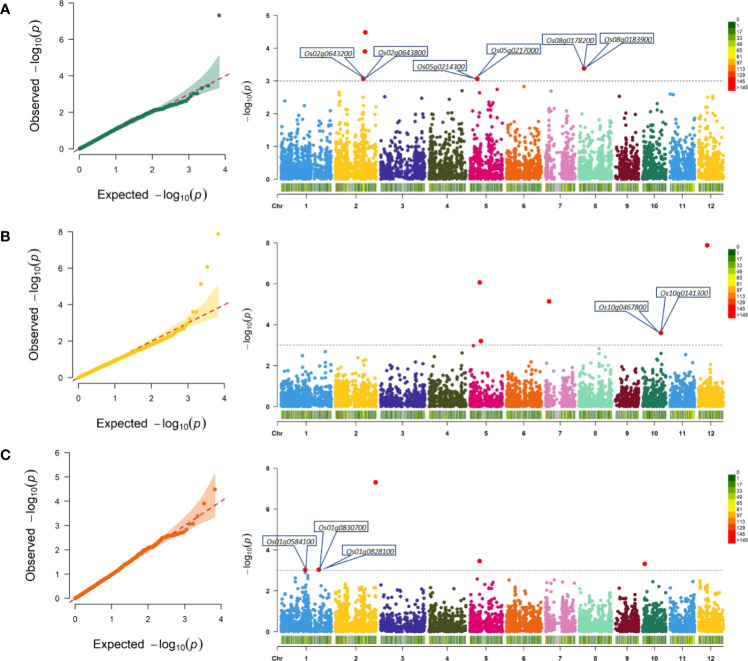
Quantile-quantile (Q–Q) plots and Manhattan plots for genomic regions associated with culm strength traits. Q–Q plots showing deviation of observed −log_10_ *p* values and expected −log_10_ *p* values, indicating the significant associations of the SNP with the trait. In Manhattan plots, chromosomes are represented on the x-axis explaining chromosome-wise SNP distribution, and significant associations at −log_10_ *p* values are represented on the y-axis. **(A)** Section modulus (SM), **(B)** bending stress (BS), and **(C)** breaking resistance as a measure of internode breaking weight (IBW). The threshold to select significant MTAs is represented as a black dotted horizontal line at LOD score 3. The putative candidate genes associated with lodging resistance are marked in the vicinity of significant MTAs in the Manhattan plots. SNPs, single-nucleotide polymorphisms; MTAs, marker–trait associations; LOD, logarithm of the odds.

Genotyping data of 6,822 SNPs and two-season phenotypic data of three culm strength traits were subjected to association analysis using a multi-locus model of BLINK. The Q–Q plots depicted less deviation of the observed *p*-values from the expected *p*-values and were therefore chosen as the best fit (**Figure 5**). At *p* ≤ 0.0001 (−log_10_ *p* ≥ 4), 15 highly significant MTAs across seven out of 12 chromosomes were identified for the three culm strength traits (**Table 2**). Two MTAs (*qSM2.1* and *qBS12.1*) showed a major effect with a phenotypic variance explained (*PVE*) of more than 10%, and the remaining 13 MTAs showed a minor effect with a *PVE* of less than 10%.

**Table 2:** Significant marker–trait associations for culm strength traits at *p* ≤ 0.0001 (−log_10_ *p* ≥ 4) were identified using the multi-locus BLINK model.

Trait	QTL	SNP	Allele		Chr	Position	*p*-Value	*a_i_*	*PVE*%
			Favorable	Alternate					
SM	*qSM1.1*	M476	C	T	1	22623828	9.31 × 10^−4^	4.05	4.79
SM	*qSM1.2*	M686	A	C	1	35313963	9.19 × 10^−4^	2.87	5.00
IBW	*qIBW2.1*	M1556	C	A	2	25884362	8.49 × 10^−4^	163.81	6.15
IBW	qIBW2.2*	M1648	T	C	2	27446861	1.26 × 10^−4^	87.76	7.85
IBW	qIBW2.3*	M1659	C	G	2	27798889	3.29 × 10^−5^	91.99	8.63
SM	qSM2.1*	M1800	G	T	2	37491093	4.86 × 10^−8^	2.39	21.87
IBW	*qIBW5.1*	M2949	T	C	5	7102952	8.60 × 10^−4^	100.90	5.89
SM	qSM5.1*	M2978	T	C	5	9207987	3.49 × 10^−4^	2.43	4.68
BS	qBS5.1*	M2985	C	T	5	9349478	8.61 × 10^−7^	4.36	6.74
BS	qBS5.2*	M3011	A	G	5	10452567	6.30 × 10^−4^	6.16	4.64
BS	qBS7.1*	M3964	T	C	7	3423329	7.30 × 10^−6^	4.94	8.30
IBW	*qIBW8.1*	M4481	G	A	8	4674600	4.15 × 10^−4^	84.39	6.99
SM	qSM10.1*	M5367	T	C	10	2185198	4.78 × 10^−4^	1.58	5.19
BS	*qBS10.1*	M5640	G	A	10	17146356	2.53 × 10^−4^	2.67	4.32
BS	qBS12.1*	M6498	A	G	12	8010763	1.32 × 10^−8^	4.61	10.14

The asterisk indicates MTAs not reported in prior studies.

SNP, single-nucleotide polymorphism; R^2^, percentage variation explained; a_i_, the additive effect of the favorable allele; SM, section modulus; IBW, internode breaking weight; QTL, quantitative trait locus; MTAs, marker–trait associations.

On further reducing the threshold to *p* ≤ 0.001 (−log_10_ *p* ≥ 3), a total of 198 MTAs were identified, corresponding to 78, 41, and 79 associations for section modulus, bending stress, and internode breaking weight, respectively. Several overlapping association signals with similar effects and sizes were observed. In earlier reports, a locus was defined as a chromosomal region at which adjacent pairs of associated SNPs were less than a certain physical distance without considering varying linkage disequilibrium levels (Yang et al., 2012). We considered adjacent SNPs spanning less than 300 kb as a single locus to reduce the redundancy of association signals similar to the previous reports (Huang et al., 2010; Chen et al., 2014; Xie et al., 2015; Guo et al., 2018; Guo et al., 2021) for identifying overlapping MTAs. After eliminating the redundant signals, 126 associations between 114 lead SNPs were identified (**Supplementary Table S3**), corresponding to 97 loci and 50, 31, and 45 MTAs for section modulus, bending stress, and internode breaking weight, respectively. MTAs were found on all the chromosomes, and chromosome 2 had the highest number of MTAs (23) followed by chromosome 5 (20 MTAs), chromosome 1 (15 MTAs), chromosome 12 (14 MTAs), chromosome 7 (12 MTAs), chromosomes 3 and 10 (11 MTAs each), chromosome 4 (10 MTAs), chromosomes 8 and 11 (7 MTAs each), and chromosome 6 (6 MTAs) (**Supplementary Table S3**). Manhattan plots depicting the significant association signals at the threshold *p* ≤ 0.001 (−log_10_ *p* ≥ 3) are provided in **Figure 5**.

A total of 50 MTAs were identified for section modulus, of which five were highly significant at *p* ≤ 0.0001 (−log_10_ *p* ≥ 4) (**Figure 5A**; **Table 2**), and the remaining 45 were significant at *p* ≤ 0.001 (−log_10_ *p* ≥ 3) (**Supplementary Table S3**). A single major effect QTL, *qSM2.1* on chromosome 2, explained a maximum *PVE* of 21.87% significant at *P_BLINK_* = 4.86 × 10^−8^ and an additive effect of 2.39 mm^3^ on the trait (**Table 2**). The highest additive effect of 4.23 mm^3^ for section modulus was identified with an association at *snp*M4010 (*P_BLINK_* = 2.9 × 10^−3^) on chromosome 7 (**Supplementary Table S3**).

For bending stress, four MTAs highly significant at *p* ≤ 0.0001 (−log_10_ *p* ≥ 4) (**Figure 5B** and **Table 2**) and 27 MTAs significant at *p* ≤ 0.001 (−log_10_ *p* ≥ 3) were identified (**Supplementary Table S3**). *qBS12.1* is a major effect of QTL at *snp*M6498 on chromosome 12, significant at *P_BLINK_* = 1.32 × 10^−8^, with a *PVE* value of 10.14% and an additive effect of 4.61. It also colocalized the section modulus, which had an additive effect of 1.24 mm^3^ (**Supplementary Table S3**). Within the same locus, an association of internode breaking weight was found in close proximity of 126 bp at *snp*M6497 with an additive effect of 67.36 g and a *PVE* value of 4.02% (**Table 2**) (**Supplementary Table S3**). Further, the highest additive effect of 7.4 was identified for bending stress at *snp*M401 on chromosome 1 (**Supplementary Table S3**).

A total of 50 MTAs were identified for internode breaking weight, of which four were highly significant at *p* ≤ 0.0001 (−log_10_ *p* ≥ 4) (**Figure 5C**; **Table 2**), and the remaining 41 were significant at *p* ≤ 0.001 (−log_10_ *p* ≥ 3) (**Supplementary Table S2**). For internode breaking weight, the significant MTA, *qIBW2.3* (*P_BLINK_* = 3.29 × 10^−5^) on chromosome 2 at *snp*M1659, had a *PVE* value of 8.63% and an additive effect of 91.99 g (**Table 2**). It also colocalized the section modulus with an additive effect of 1.24 mm^3^ (**Supplementary Table S3**). The highest additive effect of 208.41 g was identified at *snp*M3981 on chromosome 7 for internode breaking weight, with a *PVE* value of 5.64% (**Supplementary Table S3**).

**Figure 6:**
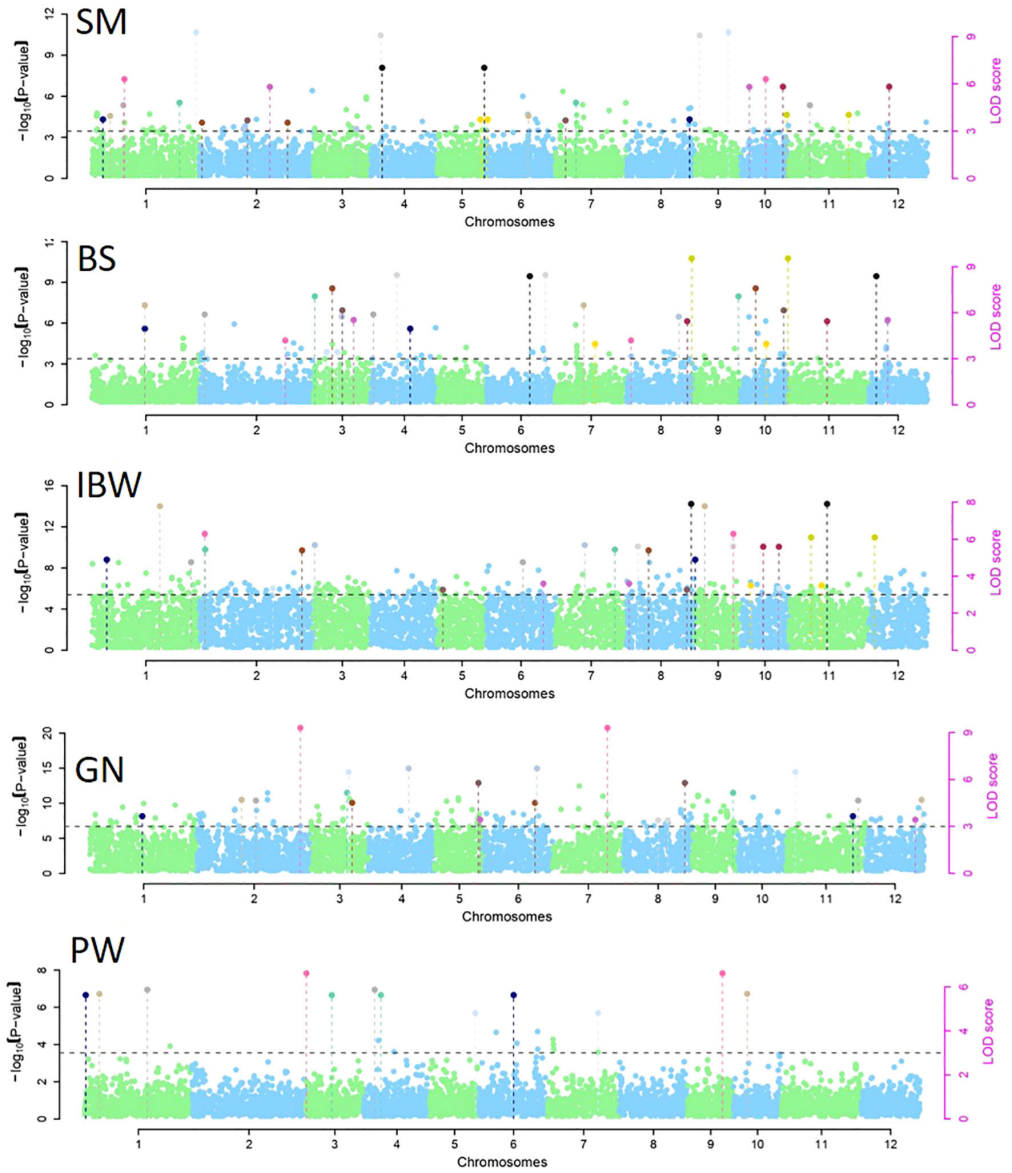
Manhattan plots for significantly associated genome-wide epistatic (SNP–SNP) interactions for section modulus (SM), bending stress (BS), breaking resistance as a measure of internode breaking weight (IBW), grain number (GN), and panicle weight (PW). SNP, single-nucleotide polymorphism.

Tests for epistatic interactions were performed using a linear regression method implemented with PLINK v1.07. Manhattan plots depicting significantly associated genome-wide epistatic (SNP–SNP) interactions for CS and GY are presented in **Figure 6**. Epistatic tests identified three and one SNP–SNP interactions associated with SM and IBW, respectively (**Supplementary Table S4**). For SM, three significant SNPs (M715, M1454, and M6514) on chromosomes 1, 2, and 12 interacted with three SNPs (M3955, M5372, and M5616) on other chromosomes 7, 10, and 10, respectively. For IBW, the SNP (M5613) on chromosome 10 exhibited significant interactions with another SNP (M5485) on the same chromosome. However, there were no epistatic interactions among the 15 highly significant MTAs presented in **Table 3**. The epistatic interactions noted with the reduction of threshold value *p* ≤ 0.001 for MTAs of IBW and SM are likely due to numerous contributing loci for the complex culm strength trait, generally with small effect.

**Table 3:** Colocalization of MTAs with previously reported putative candidate genes related to lodging resistance.

MTA	Locus ID	Position Start–end (bp)	Distance from MTA (kb)	Description
*qSM1.1*	Os01g0584100	22677778.22678619	54.79	Similar to hydroxyproline-rich glycoprotein family proteins
*qSM1.2*	Os01g0828100	35431411.35432857	117.45	Cinnamoyl-CoA reductase
*qSM1.2*	Os01g0830700	35538475.35544326	224	Xylan *O*-acetyltransferase 1
*qIBW2.1*	Os02g0643200	25839537.25842582	44.83	Transcription factor, DNA-binding intermediate protein for SLR1, modulation of the gibberellin signaling pathway, regulation of plant growth and development
*qIBW2.1*	Os02g0643800	25878905.25879875	5.46	Auxin-responsive protein
*qIBW5.1*	Os05g0214300	7082883.7085033	17.92	Bidirectional sugar transporter, gibberellin, and glucose transporter, energy source during germination and at the early stages of seedling growth
*qIBW5.1*	Os05g0217000	7219354.7219947	116.4	Arabinogalactan protein 23
*qIBW8.1*	Os08g0178200	4581432.4583489		Monosaccharide transporter 5, early flower development
*qIBW8.1*	Os08g0183900	4897613.4900341	223	Cinnamoyl-CoA reductase
*qBS10.1*	Os10g0467800	17262050.17266561	115	Secondary wall-specific cellulose synthase, secondary cell wall formation
*qBS10.1*	Os10g0141300	17262050.17266561	338.77	Wall-associated kinases

MTAs, marker–trait associations.

### 3.5 Colocalization of MTA for culm strength and yield traits

Based on the significant correlations observed between culm strength traits and yield traits in the present investigation (**Figure 2B**), we explored the genetic bases of loci that synergistically enhance culm strength and yield. Culm strength traits correlated positively with grain number and panicle weight and negatively with panicle number (**Figure 2B**); thus, we considered grain number and panicle weight for GWAS along with culm strength traits. In total, we identified 16 grain yield loci associated with culm strength traits, including seven loci for grain number and nine loci for panicle weight (**Table 4**). Each locus harbored one-grain yield trait and one to three culm strength traits on seven out of 12 chromosomes. Out of 16 associations between grain yield and culm strength traits, chromosome 2 had three clusters; chromosomes 1, 4, 5, 7, 11, and 12 had two clusters each; chromosome 8 had a single cluster.

**Table 4:** Genetic loci linking culm strength and yield traits at *p* ≤ 0.001 (−log_10_ *p* ≥ 3).

S. no.	Trait	SNP	Chr	Position	*p*-Value	*a_i_*	*PVE* %	Distance (kb)
1	GN	M496	1	23066509	9.2 × 10^−3^	21.76	3.66	169.28
	SM	M512	1	23235789	5.2 × 10^−3^	1.15	3.20	
2	IBW	M839	1	46125723	8.2 × 10^−3^	87.21	3.78	0
	PW	M839	1	46125723	1.96 × 10^−3^	0.53	4.82	
3	IBW	M1302	2	10783094	7.62 × 10^−3^	69.35	3.97	93
	PW	M1311	2	10876110	5.04 × 10^−3^	0.43	4.23	
4	SM	M1454	2	22285395	7.4 × 10^−3^	1.70	3.27	85.69
	GN	M1455	2	22371083	2.2 × 10^−3^	32.15	5.15	
5	PW	M1542	2	25733122	8.59 × 10^−3^	0.42	3.70	54.49/93.67
	BS	M1544	2	25790620	5.92 × 10^−3^	3.08	3.01	
	IBW	M1553	2	25884288	3.16 × 10^−3^	123.94	4.91	
6	PW	M2641	4	18028467	9.30 × 10^−3^	0.34	3.64	13.65
	IBW	M2642	4	18042121	9.59 × 10^−3^	56.18	3.87	
7	BS	M2771	4	30556062	9.87 × 10^−3^	2.24	2.44	151
	GN	M2793	4	30707162	3.97 × 10^−3^	43.23	4.82	
8	SM	M2949	5	7102952	2.68 × 10^−3^	1.86	3.63	0/135.95
	IBW	M2949	5	7102952	8.60 × 10^−4^	100.90	5.89	
	PW	M2952	5	7238904	4.65 × 10^−3^	0.57	4.37	
9	GN	M4676	8	18470225	9.58 × 10^−3^	39.60	3.45	133
	BS	M4677	8	18603284	7.85 × 10^−3^	2.23	2.50	
10	SM	M3074	5	17815398	7.21 × 10^−3^	1.20	3.03	0.94
	IBW	M3074	5	17815398	5.32 × 10^−3^	61.02	4.36	
	PW	M3080	5	17864293	4.34 × 10^−3^	0.34	4.19	
11	PW	M3856	7	1201789	2.18 × 10^−3^	0.45	5.09	14.14/72.06
	BS	M3871	7	1215925	7.6 × 10^−3^	6.65	3.03	
	SM	M3904	7	1287983	9.31 × 10^−3^	1.20	2.38	
12	SM	M4051	7	16056368	3.74 × 10^−3^	1.79	3.55	82.2
	GN	M4064	7	16138565	6.46 × 10^−3^	15.26	3.43	
13	PW	M5734	11	2982806	9.40 × 10^−3^	0.31	3.53	46 bp^#^
	IBW	M5735	11	2982852	2.65 × 10^−3^	72.75	4.89	
14	GN	M6252	11	21166500	8.83 × 10^−3^	19.08	3.64	58.24
	SM	M6256	11	21224745	3.52 × 10^−3^	1.99	3.62	
15	IBW	M6498	12	8010763	9.38 × 10^−3^	58.62	3.63	0
	SM	M6498	12	8010763	7.2 × 10^−3^	1.24	2.87	
	BS	M6498	12	8010763	1.32 × 10^−8^	4.61	10.14	
	GN	M6498	12	8010763	2.73 × 10^−3^	16.83	4.35	
16	PW	M6616	12	13202288	1.27 × 10^−3^	0.33	5.29	41.44
	IBW	M6622	12	13243729	2.93 × 10^−8^	191.75	5.17	

SNP, single-nucleotide polymorphism; R^2^, percentage variation explained; a_i_, the additive effect of the favorable allele; SM, section modulus; IBW, internode breaking weight; BS, bending stress; PN, panicle number; GN, grain number; PW, panicle weight; MTAs, marker–trait associations.

#Distance between MTAs at the locus is in bp, while for the remaining MTAs at other loci, it is in kb.

A QTL hotspot on chromosome 12 (*snp*M6498) colocalized grain number and all three culm strength traits. It could enhance grain yield and culm strength with an additive effect of 16.83, 1.24 mm^3^, 4.61, and 67.36 g for grain number, section modulus, bending stress, and internode breaking weight, respectively. Another six associations between grain number and culm strength traits showed a synergistic relationship on the same chromosome. The clusters for grain number and section modulus were identified on chromosomes 1, 2, 7, and 11. MTA clusters for grain number and bending stress were identified on chromosomes 4 and 12.

Among the nine genomic regions colocalizing panicle weight and culm strength traits, eight showed a synergistic relationship. An association between panicle weight and internode breaking weight at *snp*M839 on chromosome 1 was negatively correlated. Positive associations between panicle weight and internode-breaking weight were identified on chromosomes 2, 4, 11, and 12. Panicle weight colocalized with both bending stress and internode-breaking weight on chromosome 2. The two genomic regions that colocalized panicle weight and two culm strength traits, section modulus, and internode breaking weight, were identified on chromosome 5. The grain number locus associated with section modulus and bending stress was identified on chromosome 7.

### 3.6 Identification of candidate genes

The genomic regions within the same locus of the identified MTAs for culm strength were analyzed *in silico* for the presence of putative candidate genes previously reported for lodging resistance. A total of 481 putative genes were found, varying from three to 54 per QTL region. Many genes identified within the QTL region were responsible for the regulation of growth and development, resistance to abiotic and biotic stresses, amino acid transport, sugar transport, phytohormone synthesis, hypothetical and expressed proteins, glycoproteins, heat shock proteins, transcriptional factors, and precursors for various biochemical and metabolic pathways. It was interesting to note that six of the 15 MTAs identified in the present investigation were found to be in the vicinity of 11 previously reported putative candidate genes for lodging resistance (**Table 3**).

## 4 Discussion

The use of genes associated with lodging resistance in addition to the dwarfing gene is a promising approach to improving lodging resistance and further increasing productivity in rice (Ookawa et al., 2010). Owing to the complex nature of the lodging resistance in rice, it is relatively difficult to map multiple genes using a biparental-derived mapping population, wherein the genes have low individual effects and are sparsely distributed in the gene pool. Gene mapping in biparental populations is limited to the variation that exists only between the two parents used for the development of the mapping population. GWAS, therefore, offers the dual advantage of analyzing the extensive trait variation among the germplasm lines and identifying several genomic regions and alleles affecting the trait (Bollinedi et al., 2020). Globally, there are only a few GWAS reports for culm strength traits (Chigira et al., 2020; Meng et al., 2021; Nomura et al., 2021; Rashid et al., 2022). Furthermore, such studies are limited to only one report (Guo et al., 2021) for genetic associations between culm strength (CS) and grain yield (GY) traits. We report for the first time in the Indian sub-continent on genome-wide associations for culm strength traits and synergistic genetic associations between culm strength and grain yield traits.

### 4.1 Population structure

**Figure 7:**
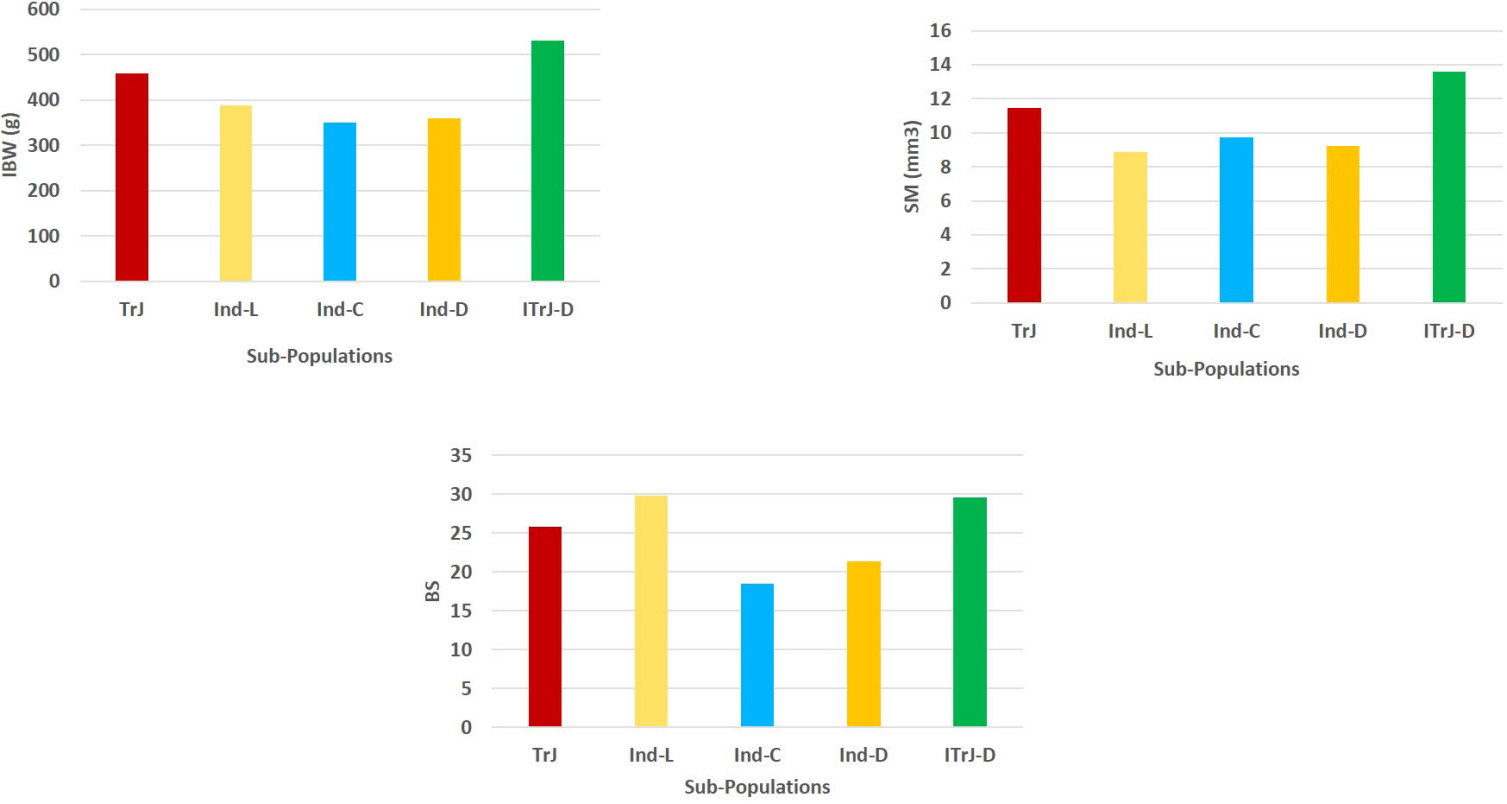
Mean phenotypic performance of the culm strength traits in the constituent groups of the GWAS panel. TrJ, tropical *japonica* accessions; Ind-L, *indica* landraces; Ind-C, *indica* cultivars; Ind-D, *indica*/*indica*-derived lines; ITrJ: *indica*/tropical *japonica*-derived lines. GWAS, genome-wide association study.

It is crucial to have control over population structure in GWAS to prevent spurious marker–trait associations (Nayak et al., 2022). The origin, selection pressure, and reproductive behavior of genotypes all have an impact on familial relatedness among individuals in an association panel (Atwell et al., 2010). In the present study, SNP-based diversity analysis provided insights into genetic relatedness among the individuals in the association panel. There was no clear grouping of *indica* and *japonica* in the population structure based on GBS data, and the two sub-populations were identified with admixtures of all the constituents of TrJ, Ind-L, Ind-C, Ind-D, and ITrJ-D (**Supplementary Table S1**). Similar to our findings, Nomura et al. (2021) also reported no clear and strong clusters in the GWAS panel for strong culm traits. The panel with a major focus on phenotypic variation was composed of culm strength traits. Population admixtures indicated no role for genetic grouping in the variation in culm strength. It is interesting to note the higher mean values for SM, BS, and IBW in ITrJ-D compared to TrJ, Ind-L, Ind-C, and Ind-D (**Figure 7**), which is similar to the findings of Nomura et al. (2019). Sub-populations arise due to allelic sharing between sub-populations, which is attributed to allelic accumulation due to spontaneous mutation over time (Agrama et al., 2007). PCA confirmed the presence of two sub-populations with a sparse distribution of alleles in the association panel. The kinship matrix generated by the VanRaden algorithm was plotted as a heatmap, showing relatedness values between 0 and +0.5, which indicated poor relationships existing between individuals in the association panel. These results assisted in understanding the population structure of the panel before proceeding to GWAS for the identification of putative genomic regions for culm strength traits. Based on the information about population structure, the multi-locus model BLINK with the EMMA approach has been selected for association analysis, which detects marker–trait associations while simultaneously addressing population structure to reduce the chances of false positives (Zhang et al., 2014; Wang et al., 2016).

### 4.2 Culm strength phenotype

High broad-sense heritability and a wide range of phenotypic variation in culm strength traits in the present study merit dissection of their genetic basis. The present investigation optimized the parameters for the measurement of culm strength in rice. Regarding the relevance of the traits for lodging resistance and considering the indefinite correlations of the lower internode length with culm-related traits for the set of genotypes in the association panel, we excluded the height traits from GWAS. With reference to the prostrate tester reading value as a measure of pushing resistance in the lower portion of the culm, it is evident that pushing resistance is more highly influenced by tiller number than the actual physical strength of the culm. For example, a strong culm genotype, IRGC 73031 (G123), identified in the present study has a pushing resistance value as low as 7, and its tiller number is only 4; however, it has the highest section modulus value (30.60 mm^3^) and highest culm diameter (7.23 mm). Hence, bending stress was measured, including both the tester reading value and tiller number, as described in Hai et al. (2005). A similar methodology was adopted in previous studies on the measurement of culm strength (Yadav et al., 2017; Jyothi et al., 2018). To further aid in assessing the culm’s physical strength, we also considered the internode breaking weight to assess the breaking resistance, an additional parameter for the culm’s physical strength. Breaking resistance (BR) as a measure of internode breaking weight is found to be a realistic parameter that depicts the true strength of the basal culm. There was a highly significant positive correlation of internode-breaking weight with other culm-related traits (**Figure 2B**), and IRGC 73031 (G123) was identified with the highest internode-breaking weight of 1,506 g (**Supplementary Table S2**).

### 4.3 Marker–trait associations and putative candidate genes

Epistasis is a genetic phenomenon of interaction that may enhance or reduce the expression (depending on degree and direction) of interacting loci underlying QTLs associated with the complex trait (Yadav et al., 2019). The role of epistasis phenomena in plant breeding has been discussed for a long time, but the extent of the expression level of quantitative traits due to this complex genetic phenomenon is poorly understood. For complex agronomic traits, estimating epistatic loci could help to clarify the complex genetic effects of GWAS loci and elucidate other types of interactions, such as genotype × environment effects (Assefa et al., 2019). Thus, there is a need to identify such epistatic gene interactions that would help to better understand the genotype–phenotype relationship of complex traits such as culm strength. The two major QTLs, *qSM2.1* and *qBS12.1*, identified in the present study with no significant epistatic interactions suggest their direct use in MAB programs.

In the present investigation, the linkage disequilibrium (LD) ranged between 200 kb and 1 Mb in the data-driven analysis for the identified MTAs. For convenience of presentation and summary of GWAS results, a locus is usually defined as a chromosomal region at which adjacent pairs of associated SNPs are less than a certain physical distance without considering varying LD levels (Yang et al., 2012). To maintain the stringency of putative candidate gene identification in the present study, the threshold was fixed at 150 kb on either side of the identified MTAs, similar to Guo et al. (2021). The 167-kb and 123-kb LD decay rates in *japonica* and *indica* subpopulations have been reported in previous studies (Huang et al., 2010; Huang et al., 2012), and Rashid et al. (2022) followed a threshold criterion of at least three SNPs in a 170-kb LD block to define the QTLs and enhance the power of GWAS resolution. Notably, six MTAs were found in the vicinity of 11 previously reported putative candidate genes for lodging resistance in the present investigation. Among them, a putative candidate gene similar to the hydroxyproline-rich glycoprotein family protein was identified to be colocalized with *qSM1.1* in close proximity to 54.79 kb. These proteins are important structural components of plant cell walls, as they can form a continuous glyco-network with non-cellulosic polysaccharides via covalent bonds or non-covalent interactions, thus strongly contributing to cell wall architecture (Hijazi et al., 2014). In the genomic region of *qSM1.2*, cinnamoyl-CoA reductase (*OsCCR6*) was identified at a distance of 117.45 kb. *OsCCR6* is a key enzyme in lignin biosynthesis (Zhao et al., 2021). Lignin is the main structural component of vascular plants’ secondary cell wall, which is related not only to plant growth and development but also to mechanical strength. Further, *qSM1.2* was found at a distance of 224 kb from the putative candidate gene regulating the synthesis of xylan *O*-acetyltransferase 1 (*OsXOAT1*). Acetylated xylans are the principal hemicelluloses in the cell walls of grass species (Qaseem and Wu, 2020). In the close vicinity of *qIBW2.1*, genes responsible for modulation of the gibberellin signaling pathway at a distance of 44.83 kb and a gene for auxin-responsive protein at 5.46 kb were identified, which have an effect on lodging resistance in rice. Auxin prompts the expression of genes related to lignin biosynthetic peroxidase (Prx) in *Zinnia elegans* and secondary growth/lignification (Gutiérrez et al., 2009). The effect of the plant hormone GA on lodging resistance in rice and increased total biomass signifies the positive impact of overexpression of GA on lodging resistance due to increased culm diameter and lignin deposition (Okuno et al., 2014). Two putative candidate genes associated with lodging resistance were identified in the *qIBW5.1* locus. Genes regulating gibberellin and glucose transport (*OsSWEET3a*) at 17.92 kb and a gene for arabinogalactan protein 23 (*OsAGP23*) (Hijazi et al., 2014) at 116.4 kb were identified in the vicinity of *qIBW5.1*. In the locus harboring *qIBW8.1*, cinnamoyl-CoA reductase (*OsCCR29*) at 223 kb was identified. The MTA *qBS10.1* was in close proximity (115 kb) to the known candidate gene cellulose synthase A7 (*OsCesA7*) on chromosome 10. *OsCesA7* is mainly responsible for cellulose synthase (UDP-forming) activity, regulating the cellulose biosynthetic process and secondary cell wall formation, thus playing a major role in enhancing stem strength (Tanaka et al., 2003; Wang et al., 2010; Kotake et al., 2011; Noda et al., 2015; Fan et al., 2017). The same locus also harbored a gene for wall-associated kinases (*OsWAK100*) at a distance of 338.77 kb, which is known to regulate secondary cell wall thickening (Ma et al., 2022). The putative candidate genes identified in the present study further need expression-based validation and functional characterization. Such characterized candidate genes may be beneficial to enhance culm strength, leading to lodging resistance in rice.

Deciphering the relationship between lodging resistance and yield traits is crucial to increasing crop productivity. In crop breeding, relationships among traits should be considered to enhance desirable correlated traits and simultaneously reduce undesirable trade-offs (Chen and Lubberstedt, 2010). To our knowledge, few studies have focused on genetic associations between culm strength and yield traits (Ookawa et al., 2010; Wang et al., 2016; Guo et al., 2021). Among the genetic associations between culm strength and grain yield traits identified in the present study, except for one negative association between internode breaking weight and panicle weight on chromosome 1, the remaining 15 associations between culm strength and panicle weight or grain number were synergistic. Some of them were colocalized with the same SNP, and some of them were within the close vicinity of 46 bp to 169.28 kb. The same QTL hotspot on chromosome 12 harboring all the culm strength traits (section modulus, bending stress, and internode breaking weight) at *snp*M6498 (pos 8010763) also harbors grain number. Also, it is a novel MTA. Internode breaking weight and grain number colocalized on chromosome 11 at a close proximity of 46 bp. Another colocalization of culm strength traits, section modulus, and internode breaking weight with panicle weight was identified on chromosome 5 at a proximal distance of 0.94 kb. Previously, significant phenotypic correlations between culm strength and yield traits were observed, and 63 loci linking them were detected (Guo et al., 2021). Interestingly, we also identified a putative candidate gene, rice floricaula/aberrant panicle organization 2 (*APO2/RFL*), linked to the MTA colocalizing BS and GN on chromosome 4 at a close proximity of 373.47 kb. *APO2/RFL* is a probable transcription factor controlling inflorescence, flower development, and short and solid culm, thus enhancing both grain yield and lodging resistance (Wang et al., 2016). QTL *SCM2*, a weaker allele of *APO1*, increases spikelet number without reducing panicle number (Ookawa et al., 2010). These colocalized MTAs can be targeted for the simultaneous improvement of culm strength and grain yield traits in *indica* rice, and favorable alleles of the major effect loci associated with only culm strength can be introgressed in the elite high-yielding *indica* cultivar background.

### 4.4 Elite donors

Culm morphological and physical strength differences in *indica* and temperate *japonica* were reported earlier; the section modulus of *indica* was greater than that of *japonica*, and the bending stress of *indica* was inferior to that of *japonica* cultivars (Ookawa et al., 2010). Superior alleles of STRONG CULM 1 and 2 (*SCM1* and *SCM2*) QTLs, which increase culm strength, were detected in an *indica* variety Habataki (Ookawa et al., 2010); similarly, *SCM3* and *SCM4* QTLs were detected in a tropical *japonica* variety Chugoku 117 (Yano et al., 2015; Yang et al., 2023). Although we observed genetic and phenotypic variations for culm strength traits in the *indica*, tropical *japonica*, and breeding lines, we also identified genotypes with superior trait values for both culm morphology and physical strength traits in both *indica* and tropical *japonica*, including their derived lines. The present study identified three genotypes, IRGC 73031 (G123), JBB 4547 (G61), and JBB 4514 (G14), with high values for all the culm strength traits (section modulus, bending stress, and internode breaking weight) and panicle weight (**Supplementary Table S2**). Additionally, 11 genotypes were identified with high values for one to four traits. Among the genotypes with superior value for all the culm strength traits, G123 is a tropical *japonica* accession, while G14 is an *indica* line, suggesting the presence of superior alleles for wider culms in *japonica* and the presence of superior alleles for physical strength in *indica* genotypes. G61 is a derived line possibly combining the superior trait values for culm morphology and physical strength from *indica* and *japonica*. Nomura et al. (2019) reported very high culm strength in Takanari, a derived line from the cross of *indica* and temperate *japonica*. Further, out of the aforementioned 14 strong culm genotypes, JBB 4514 (G14), JBB 6436 (G165), and JBB 1120 (G20) were identified with a high panicle weight of more than 5 g and G36 with a high grain number (**Supplementary Table S2**). The novel donors identified in the present study with beneficial alleles for enhanced culm strength and high yield can be utilized in the simultaneous improvement of yield and lodging resistance in rice.

## 5 Conclusion

We report for the first time GWAS for lodging resistance traits and synergistic associations for culm strength and grain yield from the Indian subcontinent. The two novel QTLs, *qSM2.1* for section modulus and *qBS12.1* for bending stress, with a major effect identified in the present study, can be further validated and employed in marker-assisted breeding for improvement of lodging resistance in elite high-yielding lodging-prone cultivars. We propose that the validation and introgression of a novel genomic region on chromosome 12, which is a QTL hotspot for all culm strength traits (section modulus, bending stress, and internode breaking weight) and grain number, can serve a dual purpose of enhancing culm strength and grain yield. MTAs found in the vicinity of previously reported putative candidate genes for lodging resistance would provide scope for further research on the biochemical basis of culm strength.

## Data availability statement

The datasets presented in this study can be found in online repositories. The names of the repository/repositories and accession number(s) can be found below: https://www.ncbi.nlm.nih.gov/sra/PRJNA1026065.

## Author contributions

JB: Conceptualization, Data curation, Funding acquisition, Investigation, Methodology, Project administration, Resources, Supervision, Validation, Visualization, Writing – original draft, Writing – review & editing. RP: Data curation, Formal analysis, Investigation, Software, Writing – review & editing. AC: Data curation, Formal analysis, Software, Writing – review & editing. AM: Writing – review & editing. SI: Investigation, Writing – review & editing. SA: Resources, Writing – review & editing. RS: Funding acquisition, Project administration, Resources, Writing – review & editing.
