# Supplementary material for: Genome-wide association studies for a comprehensive understanding of the genetic architecture of culm strength and yield traits in rice
Source: Front Plant Sci. 2024 Jan 22;14:1298083. doi: 10.3389/fpls.2023.1298083 (PMC10839031; doi:10.3389/fpls.2023.1298083)
Supplement: Supplementary file 1 [file Table_1.docx]

**Supplementary Table S1. Sub-population wise details of the 181 genotypes of the association mapping panel**

| **Code** | **Name** | **Crosses** | Sub-group |
| --- | --- | --- | --- |
| G12 | JBB 2633 | *indica*/Tropical *japonica* lines (ITrJ-D) | POP1A |
| G13 | JBB 4418 | *indica*/Tropical *japonica* lines (ITrJ-D) | POP1A |
| G15 | JBB 3089 | *indica*/Tropical *japonica* lines (ITrJ-D) | POP1A |
| G17 | JBB 9161 | *indica*/Tropical *japonica* lines (ITrJ-D) | POP1A |
| G18 | JBB 2227 | *indica*/Tropical *japonica* lines (ITrJ-D) | POP1A |
| G19 | JBB 2611 | *indica*/Tropical *japonica* lines (ITrJ-D) | POP1A |
| G20 | JBB 1120 | *indica*/Tropical *japonica* lines (ITrJ-D) | POP1A |
| G21 | JBB 1120 | *indica*/Tropical *japonica* lines (ITrJ-D) | POP1A |
| G42 | JBB 6430 | *indica*/Tropical *japonica* lines (ITrJ-D) | POP1A |
| G44 | JBB 6244 | *indica*/Tropical *japonica* lines (ITrJ-D) | POP1A |
| G72 | IL-19023 | *indica/indica* lines (Ind-D) | POP1A |
| G73 | IL-19024 | *indica/indica* lines (Ind-D) | POP1A |
| G74 | IL-19026 | *indica/indica* lines (Ind-D) | POP1A |
| G105 | IL 19285 | *indica/indica* lines (Ind-D) | POP1A |
| G111 | IL-19471 | *indica/indica* lines (Ind-D) | POP1A |
| G112 | IRGC 43741 | Tropical *japonica* (TrJ) | POP1A |
| G136 | IRGC19144 | Tropical *japonica* (TrJ) | POP1A |
| G142 | IRGC 128420 | Tropical *japonica* (TrJ) | POP1A |
| G162 | JBB 6824 | *indica*/Tropical *japonica* lines (ITrJ-D) | POP1A |
| G171 | Jarava | *indica* cutivars (Ind-C) | POP1A |
| G181 | DRR Dhan 44 | *indica* cutivars (Ind-C) | POP1A |
| G6 | Swarna | *indica* cutivars (Ind-C) | POP1B |
| G8 | RMS 2097 | *indica/indica* lines (Ind-D) | POP1B |
| G11 | JBB 6683 | *indica*/Tropical *japonica* lines (ITrJ-D) | POP1B |
| G14 | JBB 4514 | *indica/indica* lines (Ind-D) | POP1B |
| G16 | JBB 4914 | *indica*/Tropical *japonica* lines (ITrJ-D) | POP1B |
| G22 | JBB 2246 | *indica*/Tropical *japonica* lines (ITrJ-D) | POP1B |
| G39 | JBB 5952 | *indica*/Tropical *japonica* lines (ITrJ-D) | POP1B |
| G41 | JBB 6075 | *indica*/Tropical *japonica* lines (ITrJ-D) | POP1B |
| G43 | JBB 6239 | *indica*/Tropical *japonica* lines (ITrJ-D) | POP1B |
| G48 | JBB 6404 | *indica*/Tropical *japonica* lines (ITrJ-D) | POP1B |
| G51 | JBB 6653 | *indica*/Tropical *japonica* lines (ITrJ-D) | POP1B |
| G53 | Kasturi | *indica* cutivars (Ind-C) | POP1B |
| G55 | Rasi | *indica* cutivars (Ind-C) | POP1B |
| G57 | WGL 14 | *indica* cutivars (Ind-C) | POP1B |
| G58 | IR 64 | *indica* cutivars (Ind-C) | POP1B |
| G79 | IL-19198 | *indica/indica* lines (Ind-D) | POP1B |
| G88 | IL-19484 | *indica/indica* lines (Ind-D) | POP1B |
| G97 | IL-19103 | *indica/indica* lines (Ind-D) | POP1B |
| G101 | IL-19210 | *indica/indica* lines (Ind-D) | POP1B |
| G103 | IL-19246 | *indica/indica* lines (Ind-D) | POP1B |
| G119 | IRGC 78259 | Tropical *japonica* (TrJ) | POP1B |
| G140 | IRGC 127615 | *indica* landraces (Ind-L) | POP1B |
| G141 | IRGC 127538 | *indica* landraces (Ind-L) | POP1B |
| G144 | IRGC 128457 | Tropical *japonica* (TrJ) | POP1B |
| G155 | RMS 2329 | *indica/indica* lines (Ind-D) | POP1B |
| G167 | DRR Dhan 52 | *indica* cutivars (Ind-C) | POP1B |
| G168 | DRR Dhan 47 | *indica* cutivars (Ind-C) | POP1B |
| G170 | Swarnadhan | *indica* cutivars (Ind-C) | POP1B |
| G173 | Phalguna | *indica* cutivars (Ind-C) | POP1B |
| G174 | Mugad Sugandha | *indica* cutivars (Ind-C) | POP1B |
| G177 | DRR Dhan 55 | *indica* cutivars (Ind-C) | POP1B |
| G178 | Tulasi | *indica* cutivars (Ind-C) | POP1B |
| G31 | JBB 6739 | *indica*/Tropical *japonica* lines (ITrJ-D) | POP2A |
| G59 | JBB 4880 | *indica*/Tropical *japonica* lines (ITrJ-D) | POP2A |
| G67 | JBB 680-4 | *indica*/Tropical *japonica* lines (ITrJ-D) | POP2A |
| G69 | IL-19020 | *indica/indica* lines (Ind-D) | POP2A |
| G70 | IL-19021 | *indica/indica* lines (Ind-D) | POP2A |
| G75 | IL-19027 | *indica/indica* lines (Ind-D) | POP2A |
| G76 | IL-19181 | *indica/indica* lines (Ind-D) | POP2A |
| G77 | IL-19182 | *indica/indica* lines (Ind-D) | POP2A |
| G78 | IL-19185 | *indica/indica* lines (Ind-D) | POP2A |
| G80 | 19202 | *indica/indica* lines (Ind-D) | POP2A |
| G156 | JBB 6617 | *indica*/Tropical *japonica* lines (ITrJ-D) | POP2A |
| G157 | JBB 6626 | *indica*/Tropical *japonica* lines (ITrJ-D) | POP2A |
| G160 | JBB 6679 | *indica*/Tropical *japonica* lines (ITrJ-D) | POP2A |
| G1 | JBM 134 | Tropical *japonica* (TrJ) | POP2B |
| G2 | DRR Dhan 54 | *indica* cutivars (Ind-C) | POP2B |
| G3 | DRR Dhan 48 | *indica* cutivars (Ind-C) | POP2B |
| G4 | Improved Samba Mahsuri | *indica* cutivars (Ind-C) | POP2B |
| G5 | Samba Mahsuri | *indica* cutivars (Ind-C) | POP2B |
| G7 | RMS 2077 | *indica/indica* lines (Ind-D) | POP2B |
| G9 | RMS 2509 | *indica/indica* lines (Ind-D) | POP2B |
| G10 | JBB 6607 | *indica*/Tropical *japonica* lines (ITrJ-D) | POP2B |
| G23 | JBB 6874 | *indica*/Tropical *japonica* lines (ITrJ-D) | POP2B |
| G24 | JBB 6900 | *indica*/Tropical *japonica* lines (ITrJ-D) | POP2B |
| G25 | JBB 6908 | *indica*/Tropical *japonica* lines (ITrJ-D) | POP2B |
| G26 | JBB 4414 | *indica*/Tropical *japonica* lines (ITrJ-D) | POP2B |
| G27 | JBB 6631 | *indica*/Tropical *japonica* lines (ITrJ-D) | POP2B |
| G28 | JBB 6656 | *indica*/Tropical *japonica* lines (ITrJ-D) | POP2B |
| G29 | JBB 6671 | *indica*/Tropical *japonica* lines (ITrJ-D) | POP2B |
| G30 | JBB 6730 | *indica*/Tropical *japonica* lines (ITrJ-D) | POP2B |
| G32 | JBB 6787 | *indica*/Tropical *japonica* lines (ITrJ-D) | POP2B |
| G33 | JBB 6909 | *indica*/Tropical *japonica* lines (ITrJ-D) | POP2B |
| G34 | JBB 6935 | *indica*/Tropical *japonica* lines (ITrJ-D) | POP2B |
| G35 | JBB 2247 | *indica*/Tropical *japonica* lines (ITrJ-D) | POP2B |
| G36 | JBB 4513 | *indica*/Tropical *japonica* lines (ITrJ-D) | POP2B |
| G37 | JBB 6192 | *indica*/Tropical *japonica* lines (ITrJ-D) | POP2B |
| G38 | JBB 6106 | *indica*/Tropical *japonica* lines (ITrJ-D) | POP2B |
| G40 | JBB 6463 | *indica*/Tropical *japonica* lines (ITrJ-D) | POP2B |
| G45 | JBB 6440 | *indica*/Tropical *japonica* lines (ITrJ-D) | POP2B |
| G46 | JBB 6498 | *indica*/Tropical *japonica* lines (ITrJ-D) | POP2B |
| G47 | JBB 6403 | *indica*/Tropical *japonica* lines (ITrJ-D) | POP2B |
| G49 | JBB 6460 | *indica*/Tropical *japonica* lines (ITrJ-D) | POP2B |
| G50 | JBB 6616 | *indica*/Tropical *japonica* lines (ITrJ-D) | POP2B |
| G52 | JBB 6662 | *indica*/Tropical *japonica* lines (ITrJ-D) | POP2B |
| G54 | MTU 1010 | *indica* cutivars (Ind-C) | POP2B |
| G56 | Krishnahamsa | *indica* cutivars (Ind-C) | POP2B |
| G60 | JBB 3539 | *indica*/Tropical *japonica* lines (ITrJ-D) | POP2B |
| G61 | JBB 4547 | *indica*/Tropical *japonica* lines (ITrJ-D) | POP2B |
| G62 | JBB 4536 | *indica*/Tropical *japonica* lines (ITrJ-D) | POP2B |
| G63 | JB 130 | *indica*/Tropical *japonica* lines (ITrJ-D) | POP2B |
| G64 | JBB 4539 | *indica*/Tropical *japonica* lines (ITrJ-D) | POP2B |
| G65 | JB 125 | *indica*/Tropical *japonica* lines (ITrJ-D) | POP2B |
| G66 | JBB 1325 | *indica*/Tropical *japonica* lines (ITrJ-D) | POP2B |
| G68 | JBB 4888 | *indica*/Tropical *japonica* lines (ITrJ-D) | POP2B |
| G71 | IL-19022 | *indica/indica* lines (Ind-D) | POP2B |
| G81 | IL-19206 | *indica/indica* lines (Ind-D) | POP2B |
| G82 | IL-19030 | *indica/indica* lines (Ind-D) | POP2B |
| G83 | IL-19211 | *indica/indica* lines (Ind-D) | POP2B |
| G84 | IL-19247 | *indica/indica* lines (Ind-D) | POP2B |
| G85 | IL-19345 | *indica/indica* lines (Ind-D) | POP2B |
| G86 | IL-19396 | *indica/indica* lines (Ind-D) | POP2B |
| G87 | IL-19483 | *indica/indica* lines (Ind-D) | POP2B |
| G89 | IL-19001 | *indica/indica* lines (Ind-D) | POP2B |
| G90 | IL-19030 | *indica/indica* lines (Ind-D) | POP2B |
| G91 | IL-19067 | *indica/indica* lines (Ind-D) | POP2B |
| G92 | IL-19068 | *indica/indica* lines (Ind-D) | POP2B |
| G93 | IL-19088 | *indica/indica* lines (Ind-D) | POP2B |
| G94 | IL-19090 | *indica/indica* lines (Ind-D) | POP2B |
| G95 | IL-19093 | *indica/indica* lines (Ind-D) | POP2B |
| G96 | IL-19095 | *indica/indica* lines (Ind-D) | POP2B |
| G98 | IL-19147 | *indica/indica* lines (Ind-D) | POP2B |
| G99 | IL-19154 | *indica/indica* lines (Ind-D) | POP2B |
| G100 | IL-19162 | *indica/indica* lines (Ind-D) | POP2B |
| G102 | IL-19241 | *indica/indica* lines (Ind-D) | POP2B |
| G104 | IL-19249 | *indica/indica* lines (Ind-D) | POP2B |
| G106 | IL-19344 | *indica/indica* lines (Ind-D) | POP2B |
| G107 | IL-19347 | *indica/indica* lines (Ind-D) | POP2B |
| G108 | IL-19378 | *indica/indica* lines (Ind-D) | POP2B |
| G109 | IL-19379 | *indica/indica* lines (Ind-D) | POP2B |
| G110 | IL-19451 | *indica/indica* lines (Ind-D) | POP2B |
| G113 | IRGC 50448 | Tropical *japonica* (TrJ) | POP2B |
| G114 | JB 134 | Tropical *japonica* (TrJ) | POP2B |
| G115 | IRGC 18021 | Tropical *japonica* (TrJ) | POP2B |
| G116 | IRGC 50448 | Tropical *japonica* (TrJ) | POP2B |
| G117 | IRGC 33130 | Tropical *japonica* (TrJ) | POP2B |
| G118 | IRGC 29772 | Tropical *japonica* (TrJ) | POP2B |
| G120 | IRGC 74607 | Tropical *japonica* (TrJ) | POP2B |
| G121 | IRGC 74554 | Tropical *japonica* (TrJ) | POP2B |
| G122 | IRGC 73054 | Tropical *japonica* (TrJ) | POP2B |
| G123 | IRGC 73031 | Tropical *japonica* (TrJ) | POP2B |
| G124 | IRGC 71559 | Tropical *japonica* (TrJ) | POP2B |
| G125 | IRGC 68694 | Tropical *japonica* (TrJ) | POP2B |
| G126 | IRGC 67431 | Tropical *japonica* (TrJ) | POP2B |
| G127 | IRGC 66630 | Tropical *japonica* (TrJ) | POP2B |
| G128 | IRGC 64911 | Tropical *japonica* (TrJ) | POP2B |
| G129 | IRGC 64850 | Tropical *japonica* (TrJ) | POP2B |
| G130 | IRGC 62172 | Tropical *japonica* (TrJ) | POP2B |
| G131 | KJE-69 | Tropical *japonica* (TrJ) | POP2B |
| G132 | KJE-74 | Tropical *japonica* (TrJ) | POP2B |
| G133 | IRGC 62162 | Tropical *japonica* (TrJ) | POP2B |
| G134 | IRGC 60310 | Tropical *japonica* (TrJ) | POP2B |
| G135 | IRGC 57184 | Tropical *japonica* (TrJ) | POP2B |
| G137 | IRGC 73054 | Tropical *japonica* (TrJ) | POP2B |
| G138 | PTB 33 | *indica* landraces (Ind-L) | POP2B |
| G139 | IRGC 126980 | *indica* landraces (Ind-L) | POP2B |
| G143 | IRGC 125688 | Tropical *japonica* (TrJ) | POP2B |
| G145 | Rathuheenati | *indica* landraces (Ind-L) | POP2B |
| G146 | IRGC 132403 | *indica* landraces (Ind-L) | POP2B |
| G147 | IRGC 126270 | *indica* landraces (Ind-L) | POP2B |
| G148 | RMS 2075 | *indica/indica* lines (Ind-D) | POP2B |
| G149 | RMS 2084 | *indica/indica* lines (Ind-D) | POP2B |
| G150 | RMS 2085 | *indica/indica* lines (Ind-D) | POP2B |
| G151 | RMS 2092 | *indica/indica* lines (Ind-D) | POP2B |
| G152 | RMS 2301 | *indica/indica* lines (Ind-D) | POP2B |
| G153 | RMS 2304 | *indica/indica* lines (Ind-D) | POP2B |
| G154 | RMS 2309 | *indica/indica* lines (Ind-D) | POP2B |
| G158 | JBB 6627 | *indica*/Tropical *japonica* lines (ITrJ-D) | POP2B |
| G159 | JBB 6659 | *indica*/Tropical *japonica* lines (ITrJ-D) | POP2B |
| G161 | JBB 6703 | *indica*/Tropical *japonica* lines (ITrJ-D) | POP2B |
| G163 | JBB 4467 | *indica*/Tropical *japonica* lines (ITrJ-D) | POP2B |
| G164 | JBB 6411 | *indica*/Tropical *japonica* lines (ITrJ-D) | POP2B |
| G165 | JBB 6436 | *indica*/Tropical *japonica* lines (ITrJ-D) | POP2B |
| G166 | JBB 6443 | *indica*/Tropical *japonica* lines (ITrJ-D) | POP2B |
| G169 | Mandya Vijaya | *indica* cutivars (Ind-C) | POP2B |
| G172 | Akshaydhan | *indica* cutivars (Ind-C) | POP2B |
| G175 | DRR Dhan 50 | *indica* cutivars (Ind-C) | POP2B |
| G176 | DRR Dhan 53 | *indica* cutivars (Ind-C) | POP2B |
| G179 | Vikramarya | *indica* cutivars (Ind-C) | POP2B |
| G180 | DRR Dhan 42 | *indica* cutivars (Ind-C) | POP2B |

Note: The list of the genotypes in Supplementary Table S1 is in accordance with the kinship matrix presented in Figure 4.

**Supplementary Table S2.** **Mean phenotypic data of culm strength and yield traits among 181 genotypes of the association panel**

| **Code** | **PH (cm)** | **IL (cm)** | **TN** | **PN** | **GN** | **PW (g)** | **TR** | **CD (mm)** | **CT (mm)** | **IBW (g)** | **BS** | **SM (mm^3^)** |
| --- | --- | --- | --- | --- | --- | --- | --- | --- | --- | --- | --- | --- |
| G1 | 96 | 16.35 | 15 | 15 | 139 | 2.81 | 17.62 | 5.01 | 0.82 | 241 | 31.48 | 8.45 |
| G2 | 105 | 16.15 | 10 | 10 | 116 | 3.16 | 15.25 | 6.07 | 1.06 | 324 | 43.29 | 14.19 |
| G3 | 76 | 8.09 | 16 | 14 | 129 | 2.26 | 9.25 | 4.1 | 1.24 | 255 | 13.41 | 6.42 |
| G4 | 62 | 10.7 | 18 | 18 | 101 | 2.09 | 9.75 | 3.29 | 0.55 | 176 | 12.64 | 2.54 |
| G5 | 77 | 15.43 | 17 | 16 | 126 | 1.75 | 11.87 | 4.72 | 0.91 | 371 | 17.86 | 8.2 |
| G6 | 68 | 10.87 | 17 | 17 | 118 | 2.02 | 11.38 | 4.71 | 0.98 | 188 | 16.86 | 7.95 |
| G7 | 86 | 16.87 | 13 | 13 | 238 | 3.61 | 11.94 | 6.32 | 1.26 | 621 | 24.06 | 19.42 |
| G8 | 112 | 14.8 | 8 | 7 | 259 | 5.41 | 14.88 | 6.39 | 1.05 | 789 | 44.4 | 18.32 |
| G9 | 78 | 12.85 | 10 | 9 | 176 | 3.19 | 10.47 | 5.21 | 1.15 | 776 | 27.16 | 12.88 |
| G10 | 88 | 14.01 | 16 | 16 | 211 | 4.51 | 14.13 | 5.39 | 1.17 | 371 | 23.57 | 12.77 |
| G11 | 86 | 11.66 | 15 | 14 | 165 | 5.06 | 14.63 | 5.06 | 1.29 | 524 | 24.82 | 10.41 |
| G12 | 98 | 10.96 | 18 | 16 | 167 | 3.31 | 14.13 | 5.43 | 1.43 | 436 | 19.78 | 13.95 |
| G13 | 101 | 11.46 | 13 | 13 | 157 | 2.76 | 14.63 | 6.37 | 1.37 | 611 | 28.01 | 21.04 |
| G14 | 90 | 11.76 | 9 | 9 | 149 | 5.51 | 20.38 | 6.95 | 1.34 | 876 | 59.76 | 26.19 |
| G15 | 109 | 14.01 | 10 | 10 | 209 | 4.06 | 18.13 | 6.28 | 1.12 | 819 | 45.12 | 18.14 |
| G16 | 87 | 15.51 | 15 | 15 | 146 | 2.31 | 22.13 | 5.48 | 1.09 | 251 | 36.79 | 12.55 |
| G17 | 98 | 6.48 | 11 | 10 | 162 | 3.41 | 16.13 | 5.17 | 0.95 | 251 | 36.58 | 10.27 |
| G18 | 104 | 15.76 | 15 | 15 | 193 | 4.91 | 11.13 | 6.36 | 1.19 | 401 | 18.46 | 19.69 |
| G19 | 108 | 19.56 | 14 | 13 | 154 | 5.06 | 17.63 | 5.29 | 0.99 | 241 | 31.89 | 11.39 |
| G20 | 100 | 10.06 | 18 | 16 | 231 | 5.11 | 26.63 | 7.01 | 1.56 | 771 | 37.95 | 27.32 |
| G21 | 107 | 11.46 | 11 | 9 | 205 | 3.86 | 14.88 | 6.2 | 1.38 | 614 | 36.06 | 19.34 |
| G22 | 109 | 16.66 | 17 | 17 | 171 | 4.56 | 18.63 | 5.94 | 1.08 | 376 | 27.33 | 15.43 |
| G23 | 104 | 12.01 | 16 | 14 | 176 | 2.61 | 14.63 | 5.26 | 1.02 | 421 | 23.51 | 10.91 |
| G24 | 93 | 11.51 | 18 | 16 | 188 | 2.91 | 22.38 | 6.14 | 1.5 | 841 | 31.92 | 19.19 |
| G25 | 104 | 13.46 | 20 | 19 | 232 | 5.41 | 21.13 | 5.46 | 0.52 | 291 | 26.79 | 6.63 |
| G26 | 74 | 12.76 | 18 | 18 | 195 | 2.41 | 15.88 | 6.01 | 1.22 | 706 | 22.09 | 17.08 |
| G27 | 67 | 12.16 | 13 | 12 | 121 | 1.46 | 15.63 | 5.73 | 1.1 | 429 | 29.93 | 14.6 |
| G28 | 108 | 13.91 | 12 | 10 | 196 | 3.61 | 21.13 | 5.37 | 1.52 | 666 | 45.77 | 13.39 |
| G29 | 82 | 11.41 | 11 | 10 | 166 | 3.56 | 17.88 | 5.99 | 1.08 | 581 | 40.64 | 15.58 |
| G30 | 97 | 10.11 | 25 | 20 | 150 | 1.66 | 25.13 | 6.11 | 1.23 | 531 | 26.14 | 18.52 |
| G31 | 72 | 15.01 | 13 | 11 | 150 | 2.71 | 10.38 | 6.31 | 1.62 | 876 | 20.56 | 21.21 |
| G32 | 101 | 13.61 | 14 | 16 | 213 | 3.46 | 23.63 | 5.35 | 0.97 | 371 | 42.37 | 11.4 |
| G33 | 94 | 10.91 | 15 | 13 | 143 | 2.81 | 17.63 | 6.14 | 1.34 | 711 | 30.35 | 19.07 |
| G34 | 101 | 14.86 | 10 | 10 | 256 | 5.21 | 11.38 | 5.15 | 0.64 | 544 | 28.6 | 7.74 |
| G35 | 99 | 12.41 | 15 | 14 | 206 | 3.81 | 29.63 | 5.63 | 1.15 | 626 | 49.29 | 13.88 |
| G36 | 93 | 16.26 | 11 | 11 | 244 | 4.61 | 15.63 | 7.29 | 1.28 | 654 | 37.28 | 30.16 |
| G37 | 93 | 9.46 | 26 | 24 | 182 | 1.55 | 23.63 | 6.13 | 1.45 | 681 | 23.33 | 18.46 |
| G38 | 84 | 15.16 | 22 | 20 | 144 | 2.96 | 20.63 | 5.02 | 1.08 | 351 | 23.49 | 10 |
| G39 | 90 | 10.91 | 12 | 11 | 211 | 4.76 | 19.13 | 6.37 | 1.36 | 889 | 39.71 | 20.5 |
| G40 | 85 | 13.66 | 14 | 13 | 203 | 3.76 | 20.13 | 5.52 | 1.04 | 501 | 36.15 | 13.12 |
| G41 | 90 | 15.76 | 9 | 9 | 170 | 3.41 | 9.13 | 3.71 | 0.91 | 314 | 25.12 | 4.08 |
| G42 | 107 | 11.91 | 13 | 13 | 179 | 3.91 | 10.63 | 6.07 | 1.27 | 511 | 20.96 | 18.23 |
| G43 | 98 | 10.51 | 11 | 11 | 195 | 4.21 | 13.63 | 4.45 | 0.93 | 601 | 32.28 | 6.84 |
| G44 | 102 | 14.76 | 11 | 11 | 161 | 2.71 | 9.38 | 6.07 | 1.09 | 456 | 21.37 | 16.45 |
| G45 | 95 | 11.96 | 13 | 12 | 214 | 4.41 | 15.13 | 6.78 | 1.16 | 601 | 28.69 | 23.03 |
| G46 | 96 | 10.86 | 16 | 16 | 130 | 2.21 | 17.63 | 5.05 | 1.14 | 614 | 27.47 | 10.51 |
| G47 | 93 | 10.46 | 13 | 12 | 110 | 1.91 | 10.38 | 5.83 | 0.96 | 769 | 20.64 | 14.06 |
| G48 | 91 | 13.11 | 21 | 19 | 141 | 2.46 | 17.13 | 4.71 | 0.33 | 411 | 21.26 | 3.98 |
| G49 | 111 | 12.11 | 12 | 11 | 215 | 4.31 | 14.13 | 4.13 | 0.92 | 831 | 31.23 | 5.63 |
| G50 | 93 | 12.26 | 9 | 8 | 145 | 3.71 | 10.63 | 5 | 0.7 | 386 | 30.48 | 8.42 |
| G51 | 95 | 10.66 | 13 | 12 | 156 | 1.91 | 9.63 | 4.85 | 1.54 | 641 | 19.65 | 9.78 |
| G52 | 84 | 16.76 | 14 | 13 | 191 | 3.86 | 10.63 | 5.08 | 0.62 | 251 | 18.91 | 7.61 |
| G53 | 96 | 18.64 | 13 | 13 | 154 | 2.15 | 9.52 | 5.34 | 0.75 | 299 | 18.79 | 9.62 |
| G54 | 78 | 14.69 | 15 | 14 | 169 | 3.55 | 7.77 | 4.8 | 1.14 | 346 | 13.78 | 8.57 |
| G55 | 89 | 17.94 | 24 | 23 | 237 | 4.6 | 12.52 | 4.85 | 1.09 | 336 | 13.83 | 8.91 |
| G56 | 77 | 17.64 | 18 | 18 | 101 | 2.3 | 12.27 | 5.69 | 1.48 | 534 | 17.36 | 15.64 |
| G57 | 97 | 12.09 | 21 | 20 | 196 | 2.9 | 10.77 | 5.8 | 1.48 | 669 | 13.3 | 15.99 |
| G58 | 80 | 16.49 | 25 | 24 | 108 | 2.6 | 10.77 | 5.16 | 1.29 | 326 | 11.32 | 11.51 |
| G59 | 77 | 13.74 | 17 | 15 | 114 | 1.55 | 9.77 | 5.37 | 1.08 | 644 | 15.14 | 12.69 |
| G60 | 88 | 15.59 | 11 | 10 | 107 | 1.7 | 18.52 | 6.7 | 1.47 | 541 | 44.38 | 23.89 |
| G61 | 90 | 14.09 | 13 | 12 | 183 | 4.65 | 24.02 | 6.69 | 2.21 | 869 | 48.93 | 26.79 |
| G62 | 86 | 18.69 | 20 | 18 | 85 | 1.8 | 18.77 | 5.19 | 1.39 | 254 | 24.71 | 11.95 |
| G63 | 98 | 12.39 | 16 | 16 | 97 | 0.97 | 15.77 | 5.52 | 0.99 | 724 | 25.02 | 11.8 |
| G64 | 108 | 16.04 | 18 | 17 | 163 | 3.4 | 15.77 | 5.53 | 1.32 | 184 | 23.17 | 13.23 |
| G65 | 67 | 11.19 | 23 | 22 | 100 | 2.15 | 14.52 | 4.12 | 0.9 | 266 | 16.34 | 5.86 |
| G66 | 100 | 13.09 | 24 | 24 | 201 | 4.15 | 17.77 | 5.66 | 0.92 | 369 | 19.52 | 12.59 |
| G67 | 84 | 15.24 | 27 | 25 | 130 | 2.45 | 15.27 | 5.28 | 0.96 | 379 | 15 | 11.17 |
| G68 | 96 | 15.04 | 8 | 8 | 119 | 2.95 | 9.27 | 5.32 | 0.83 | 389 | 28.93 | 9.72 |
| G69 | 79 | 14.99 | 25 | 22 | 93 | 2.2 | 15.02 | 4.7 | 0.87 | 339 | 16.11 | 7.32 |
| G70 | 80 | 13.04 | 22 | 20 | 127 | 3.75 | 16.77 | 4.4 | 0.48 | 139 | 19.56 | 4.59 |
| G71 | 83 | 10.74 | 15 | 13 | 156 | 3.65 | 17.27 | 4.59 | 0.62 | 219 | 30.4 | 5.86 |
| G72 | 83 | 14.34 | 24 | 23 | 116 | 3.35 | 14.27 | 4.84 | 0.83 | 164 | 15.4 | 7.95 |
| G73 | 79 | 10.74 | 23 | 23 | 155 | 4.55 | 15.77 | 4.41 | 0.6 | 274 | 17.66 | 5.23 |
| G74 | 76 | 16.99 | 17 | 16 | 133 | 4 | 18.27 | 4.76 | 1.27 | 319 | 27.47 | 8.87 |
| G75 | 71 | 12.99 | 25 | 23 | 143 | 5 | 11.77 | 4.38 | 1.2 | 159 | 12.71 | 6.67 |
| G76 | 90 | 13.09 | 18 | 17 | 117 | 4.35 | 16.27 | 4.99 | 0.62 | 374 | 24.02 | 8.34 |
| G77 | 78 | 11.79 | 10 | 10 | 119 | 4 | 14.77 | 4.99 | 0.95 | 234 | 37.05 | 8.94 |
| G78 | 76 | 14.49 | 16 | 15 | 93 | 3.35 | 16.77 | 5.06 | 1.03 | 229 | 27.37 | 11.06 |
| G79 | 100 | 17.19 | 17 | 14 | 100 | 3.75 | 16.27 | 5.65 | 0.83 | 464 | 25.02 | 12.77 |
| G80 | 89 | 15.24 | 17 | 16 | 102 | 3.3 | 12.27 | 5.25 | 0.78 | 459 | 18.95 | 9.2 |
| G81 | 115 | 18.04 | 13 | 11 | 97 | 2.6 | 16.27 | 5.51 | 1.12 | 399 | 31.9 | 12.87 |
| G82 | 107 | 17.49 | 14 | 12 | 149 | 4.15 | 19.27 | 5.66 | 0.92 | 359 | 36.1 | 13.08 |
| G83 | 76 | 14.09 | 23 | 22 | 104 | 3.55 | 13.77 | 4.88 | 0.76 | 124 | 15.47 | 7.79 |
| G84 | 82 | 11.79 | 18 | 17 | 116 | 3 | 13.77 | 5.15 | 0.81 | 279 | 19.86 | 9.78 |
| G85 | 80 | 15.04 | 10 | 9 | 231 | 4.8 | 10.27 | 5.13 | 0.84 | 324 | 27.36 | 10.14 |
| G86 | 97 | 15.19 | 16 | 14 | 104 | 3.3 | 15.27 | 5.65 | 1.19 | 369 | 24.97 | 13.35 |
| G87 | 85 | 11.64 | 12 | 11 | 207 | 4.3 | 9.77 | 4.56 | 0.6 | 204 | 20.59 | 5.57 |
| G88 | 93 | 13.64 | 12 | 12 | 252 | 4.8 | 11.77 | 5.27 | 0.7 | 259 | 25.8 | 8.22 |
| G89 | 83 | 9.54 | 20 | 19 | 180 | 4.5 | 15.77 | 4.66 | 0.88 | 271 | 20.33 | 7.86 |
| G90 | 86 | 12.99 | 21 | 21 | 141 | 3.75 | 17.02 | 4.65 | 0.93 | 199 | 20.74 | 7.9 |
| G91 | 100 | 12.24 | 15 | 14 | 166 | 3.85 | 15.77 | 4.53 | 0.63 | 684 | 26.69 | 5.58 |
| G92 | 88 | 13.39 | 20 | 19 | 120 | 2.9 | 9.02 | 3.97 | 0.64 | 309 | 12.16 | 4.6 |
| G93 | 89 | 9.04 | 20 | 19 | 186 | 2.8 | 19.77 | 4.62 | 0.73 | 219 | 25.36 | 6.85 |
| G94 | 82 | 9.79 | 23 | 18 | 173 | 4.85 | 8.27 | 4.44 | 0.9 | 369 | 9.73 | 6.99 |
| G95 | 88 | 12.79 | 22 | 21 | 225 | 5.2 | 14.27 | 4.39 | 0.62 | 309 | 16.74 | 4.42 |
| G96 | 86 | 11.93 | 25 | 21 | 195 | 3.17 | 15.49 | 4.56 | 0.63 | 404 | 15.17 | 5.78 |
| G97 | 84 | 9.77 | 16 | 15 | 175 | 3.72 | 10.49 | 4.17 | 0.88 | 254 | 15.94 | 4.73 |
| G98 | 95 | 11.13 | 16 | 13 | 127 | 3.12 | 14.99 | 4.63 | 0.61 | 434 | 22.24 | 6.67 |
| G99 | 129 | 14.53 | 9 | 9 | 97 | 3.22 | 14.49 | 3.76 | 0.82 | 829 | 37.64 | 3.78 |
| G100 | 76 | 11.18 | 11 | 10 | 172 | 4.02 | 7.49 | 5.36 | 0.7 | 404 | 15.92 | 8.64 |
| G101 | 90 | 10.23 | 18 | 17 | 125 | 3.42 | 13.49 | 4.25 | 0.55 | 354 | 18.14 | 4.63 |
| G102 | 80 | 14.63 | 19 | 17 | 120 | 3.52 | 9.99 | 4.45 | 0.71 | 254 | 12.6 | 5.68 |
| G103 | 91 | 14.58 | 21 | 19 | 147 | 4.12 | 16.79 | 4.53 | 0.75 | 139 | 19.5 | 6.25 |
| G104 | 113 | 15.23 | 17 | 16 | 95 | 2.37 | 16.99 | 4.51 | 0.77 | 294 | 23.71 | 6.93 |
| G105 | 89 | 11.28 | 32 | 31 | 135 | 4.52 | 8.24 | 4.27 | 0.6 | 449 | 5.95 | 4.45 |
| G106 | 80 | 10.09 | 19 | 18 | 239 | 4.47 | 11.49 | 4.05 | 0.88 | 224 | 14.58 | 4.88 |
| G107 | 80 | 9.97 | 20 | 20 | 191 | 4.17 | 13.99 | 5.01 | 0.96 | 404 | 16.7 | 9.52 |
| G108 | 85 | 8.13 | 19 | 18 | 131 | 3.87 | 15.49 | 4.69 | 0.39 | 399 | 20.68 | 5.39 |
| G109 | 73 | 14.88 | 17 | 15 | 150 | 3.22 | 9.74 | 5.27 | 1.14 | 559 | 13.77 | 11.93 |
| G110 | 72 | 12.68 | 18 | 16 | 113 | 4.42 | 7.99 | 3.98 | 0.78 | 266 | 10.46 | 4.75 |
| G111 | 84 | 11.73 | 16 | 16 | 147 | 4.77 | 11.49 | 4.83 | 0.84 | 369 | 16.82 | 8.76 |
| G112 | 102 | 19.18 | 14 | 12 | 169 | 3.57 | 15.49 | 5.38 | 0.83 | 244 | 26.95 | 10 |
| G113 | 87 | 17.93 | 21 | 19 | 115 | 2.72 | 16.74 | 4.57 | 0.77 | 319 | 19.47 | 7.18 |
| G114 | 85 | 16.13 | 13 | 12 | 125 | 2.82 | 16.49 | 5.21 | 0.72 | 264 | 30.28 | 8.2 |
| G115 | 123 | 10.73 | 11 | 10 | 119 | 2.72 | 15.99 | 4.71 | 0.72 | 914 | 36.11 | 7 |
| G116 | 97 | 9.98 | 9 | 8 | 112 | 2.77 | 7.49 | 5.37 | 1.2 | 721 | 20.38 | 11.9 |
| G117 | 122 | 15.48 | 11 | 10 | 82 | 1.67 | 12.99 | 5.5 | 0.72 | 366 | 29.57 | 9.89 |
| G118 | 140 | 17.53 | 26 | 24 | 199 | 1.72 | 28.49 | 5.39 | 0.89 | 344 | 27.28 | 10.92 |
| G119 | 80 | 17.58 | 17 | 17 | 115 | 2.12 | 11.49 | 5.54 | 1.15 | 269 | 15.87 | 13.62 |
| G120 | 117 | 16.18 | 15 | 15 | 353 | 3.57 | 9.74 | 5.47 | 1.27 | 326 | 15.1 | 12.42 |
| G121 | 107 | 18.18 | 11 | 9 | 164 | 3.02 | 7.49 | 5.19 | 0.7 | 364 | 16.49 | 9.1 |
| G122 | 131 | 19.98 | 12 | 9 | 95 | 2.37 | 10.24 | 5.97 | 0.7 | 504 | 20 | 12.84 |
| G123 | 116 | 12.48 | 3 | 3 | 170 | 2.52 | 7.24 | 7.21 | 1.57 | 1506 | 52.05 | 30.22 |
| G124 | 98 | 9.63 | 13 | 12 | 125 | 2.87 | 10.24 | 2.68 | 0.74 | 304 | 19.39 | 1.25 |
| G125 | 115 | 17.78 | 9 | 6 | 136 | 2.72 | 10.99 | 5.37 | 0.93 | 344 | 28.49 | 11.35 |
| G126 | 87 | 10.38 | 13 | 12 | 112 | 2.17 | 11.24 | 4.48 | 0.8 | 284 | 21.08 | 6.26 |
| G127 | 88 | 10.18 | 15 | 14 | 126 | 2.37 | 5.49 | 3.98 | 0.74 | 234 | 8.48 | 4.24 |
| G128 | 125 | 13.93 | 10 | 9 | 113 | 3.02 | 20.49 | 5.87 | 1 | 824 | 50.7 | 14.35 |
| G129 | 126 | 14.98 | 15 | 13 | 113 | 1.92 | 13.49 | 5.44 | 1.01 | 594 | 21.42 | 12.07 |
| G130 | 122 | 15.33 | 17 | 13 | 154 | 2.77 | 13.99 | 5.86 | 1.22 | 809 | 20.06 | 16.19 |
| G131 | 139 | 16.18 | 10 | 9 | 100 | 2.02 | 16.49 | 5.34 | 0.83 | 229 | 40.99 | 9.94 |
| G132 | 121 | 18.88 | 18 | 14 | 100 | 2.22 | 21.99 | 6.72 | 1.27 | 679 | 29.54 | 23.41 |
| G133 | 143 | 16.38 | 9 | 9 | 145 | 3.22 | 15.49 | 6.04 | 1.18 | 304 | 40.28 | 16.49 |
| G134 | 117 | 15.03 | 9 | 8 | 127 | 4.07 | 8.99 | 5.15 | 0.93 | 494 | 23.31 | 9.71 |
| G135 | 108 | 18.38 | 16 | 14 | 158 | 3.82 | 12.99 | 6.06 | 1.07 | 629 | 19.64 | 16.25 |
| G136 | 110 | 10.98 | 16 | 14 | 162 | 3.17 | 14.74 | 5.38 | 1.27 | 454 | 21.83 | 15.47 |
| G137 | 81 | 19.38 | 16 | 16 | 80 | 1.67 | 9.49 | 4.4 | 0.72 | 119 | 13.88 | 5.58 |
| G138 | 91 | 10.68 | 18 | 15 | 103 | 2.17 | 14.99 | 4.67 | 0.64 | 561 | 20.5 | 6.21 |
| G139 | 106 | 10.87 | 17 | 16 | 193 | 1.33 | 19.61 | 4.44 | 0.01 | 381 | 28.22 | 5.64 |
| G140 | 122 | 18.52 | 17 | 16 | 154 | 3.03 | 14.36 | 5.23 | 0.52 | 366 | 21.58 | 9.47 |
| G141 | 88 | 16.67 | 21 | 17 | 107 | 1.58 | 18.36 | 4.53 | 0.58 | 303 | 21.42 | 7.76 |
| G142 | 97 | 12.52 | 20 | 18 | 102 | 1.23 | 12.61 | 4.89 | 0.52 | 261 | 15.88 | 8.39 |
| G143 | 98 | 20.87 | 3 | 2 | 122 | 1.73 | 5.11 | 5.79 | 0.72 | 391 | 39.21 | 15.17 |
| G144 | 101 | 17.62 | 23 | 21 | 129 | 3.03 | 20.11 | 4.71 | 0.8 | 328 | 24.37 | 8.81 |
| G145 | 114 | 11.47 | 5 | 4 | 183 | 1.73 | 10.11 | 4.4 | 1.07 | 406 | 54.84 | 7.02 |
| G146 | 96 | 20.22 | 13 | 13 | 96 | 1.68 | 18.61 | 5.38 | 0.68 | 216 | 34.93 | 12.04 |
| G147 | 109 | 11.22 | 28 | 12 | 142 | 1.63 | 25.11 | 5.1 | 0.78 | 573 | 23.07 | 11.64 |
| G148 | 111 | 14.77 | 19 | 19 | 410 | 5.23 | 11.11 | 4.85 | 0.7 | 596 | 14.51 | 9.92 |
| G149 | 83 | 11.32 | 18 | 16 | 143 | 3.48 | 18.11 | 5.18 | 0.61 | 441 | 24.52 | 10.72 |
| G150 | 87 | 11.77 | 14 | 13 | 135 | 2.93 | 12.61 | 5.64 | 0.82 | 551 | 22.42 | 16.35 |
| G151 | 74 | 14.52 | 9 | 8 | 216 | 4.43 | 10.11 | 4.46 | 0.49 | 206 | 28.18 | 6.64 |
| G152 | 78 | 15.87 | 15 | 14 | 201 | 2.93 | 13.11 | 5.53 | 0.95 | 428 | 22.17 | 15.88 |
| G153 | 77 | 14.47 | 19 | 17 | 109 | 1.93 | 9.86 | 6.14 | 1.01 | 348 | 12.79 | 20.51 |
| G154 | 100 | 14.57 | 13 | 12 | 194 | 3.93 | 17.11 | 5.95 | 0.68 | 458 | 33.28 | 16.96 |
| G155 | 75 | 15.37 | 14 | 14 | 192 | 4.23 | 8.61 | 5.03 | 0.56 | 196 | 15.11 | 9.59 |
| G156 | 88 | 7.67 | 10 | 10 | 117 | 2.08 | 12.11 | 5.15 | 0.74 | 341 | 29.52 | 11.92 |
| G157 | 100 | 12.72 | 14 | 14 | 251 | 4.03 | 19.61 | 5.1 | 0.64 | 531 | 35.34 | 10 |
| G158 | 93 | 9.67 | 13 | 11 | 160 | 3.43 | 13.86 | 2.79 | 0.58 | 528 | 26.07 | 1.99 |
| G159 | 72 | 12.07 | 12 | 11 | 197 | 5.33 | 5.61 | 2.68 | 0.93 | 483 | 12.13 | 1.8 |
| G160 | 96 | 13.67 | 15 | 14 | 187 | 2.83 | 14.61 | 5.22 | 0.51 | 353 | 24.98 | 9.26 |
| G161 | 92 | 10.97 | 7 | 4 | 205 | 4.73 | 8.61 | 5.19 | 0.71 | 516 | 32.96 | 11.14 |
| G162 | 73 | 11.22 | 12 | 10 | 163 | 3.38 | 11.11 | 4.87 | 0.56 | 231 | 22.84 | 9.76 |
| G163 | 90 | 10.22 | 11 | 10 | 128 | 1.73 | 15.61 | 5.55 | 0.4 | 386 | 34.19 | 9.74 |
| G164 | 86 | 12.47 | 14 | 13 | 201 | 3.33 | 19.61 | 5.21 | 0.83 | 436 | 35.34 | 12.16 |
| G165 | 97 | 10.57 | 10 | 9 | 220 | 4.83 | 15.11 | 5.92 | 1.14 | 883 | 39.73 | 19.72 |
| G166 | 100 | 11.07 | 10 | 10 | 122 | 2.58 | 9.36 | 5.9 | 0.92 | 776 | 22.93 | 16.91 |
| G167 | 94 | 11.37 | 16 | 15 | 100 | 2.03 | 16.61 | 4.67 | 0.96 | 401 | 25.32 | 9.09 |
| G168 | 94 | 20.77 | 14 | 11 | 103 | 1.98 | 20.11 | 4.42 | 0.84 | 173 | 35.09 | 7.95 |
| G169 | 111 | 11.32 | 23 | 21 | 186 | 2.78 | 13.11 | 5.15 | 0.79 | 321 | 14.49 | 11.47 |
| G170 | 88 | 13.27 | 17 | 15 | 149 | 2.68 | 12.36 | 5.77 | 0.91 | 426 | 18.38 | 16.61 |
| G171 | 89 | 11.22 | 31 | 29 | 158 | 3.23 | 23.11 | 4.46 | 0.8 | 478 | 18.27 | 8.58 |
| G172 | 94 | 8.97 | 17 | 16 | 129 | 2.18 | 13.61 | 5.06 | 1.01 | 416 | 19.61 | 12.3 |
| G173 | 88 | 15.62 | 27 | 26 | 104 | 2.23 | 14.11 | 4.86 | 0.45 | 366 | 13.16 | 7.93 |
| G174 | 84 | 10.32 | 23 | 22 | 115 | 2.08 | 18.11 | 4.91 | 0.74 | 498 | 19.35 | 10.77 |
| G175 | 83 | 14.47 | 16 | 15 | 139 | 2.08 | 14.61 | 4.03 | 0.36 | 116 | 22.55 | 4.16 |
| G176 | 73 | 12.37 | 17 | 14 | 149 | 1.48 | 11.86 | 4.58 | 1.07 | 448 | 17.64 | 9.59 |
| G177 | 87 | 14.72 | 20 | 18 | 128 | 2.53 | 15.36 | 4.68 | 0.81 | 271 | 19.32 | 9.4 |
| G178 | 79 | 15.02 | 23 | 22 | 162 | 2.33 | 11.61 | 4.51 | 0.56 | 211 | 12.45 | 7.45 |
| G179 | 91 | 11.37 | 20 | 19 | 96 | 2.53 | 16.11 | 4.62 | 0.52 | 151 | 20.34 | 7.45 |
| G180 | 88 | 13.97 | 17 | 15 | 119 | 2.28 | 12.36 | 4.64 | 0.57 | 426 | 17.85 | 8.27 |
| G181 | 101 | 11.02 | 19 | 17 | 106 | 1.93 | 9.61 | 5.29 | 0.84 | 551 | 13.11 | 12.91 |

**Supplementary Table S3 Significant marker-trait associations for culm strength traits at *P*≤0.001 (-log_10_*P*≥3) identified using multi-locus BLINK model**

| **S. No** | **Trait** | **SNP** | **Chr** | **Pos** | **P.value** | **Effect** | **PVE%** |
| --- | --- | --- | --- | --- | --- | --- | --- |
| 1 | IBW | M118 | 1 | 3665065 | 0.00409219 | 192.79 | 4.86 |
| 2 | SM | M264 | 1 | 13449740 | 0.002385327 | 1.64 | 3.94 |
| 3 | SM | M339 | 1 | 15862943 | 0.007778928 | 1.36 | 2.84 |
| 4 | BS | M401 | 1 | 19995768 | 0.003269905 | 3.15 | 2.84 |
| 5 | SM | M410 | 1 | 20342633 | 0.00701517 | 1.56 | 2.69 |
| 6 | IBW | M441 | 1 | 21895703 | 0.005726819 | 129.66 | 4.58 |
| 7 | SM | M471 | 1 | 22623828 | 0.000931168 | 3.05 | 4.79 |
| 8 | SM | M512 | 1 | 23235789 | 0.005159953 | 1.15 | 3.20 |
| 9 | SM | M561 | 1 | 25122293 | 0.001814595 | 1.57 | 3.67 |
| 10 | SM | M686 | 1 | 35313963 | 0.000919038 | 2.87 | 5.00 |
| 11 | SM | M715 | 1 | 37528027 | 0.005650648 | 1.01 | 3.12 |
| 12 | BS | M746 | 1 | 41419604 | 0.002086954 | 5.56 | 3.69 |
| 13 | SM | M752 | 1 | 41462770 | 0.009269594 | 2.16 | 2.81 |
| 14 | IBW | M839 | 1 | 46125723 | 0.008156274 | 87.21 | 3.78 |
| 15 | BS | M871 | 1 | 46559221 | 0.008930601 | 2.28 | 2.41 |
| 16 | SM | M1075 | 2 | 3628180 | 0.009944764 | 2.79 | 3.10 |
| 17 | IBW | M1075 | 2 | 3628180 | 0.002205668 | 160.65 | 5.48 |
| 18 | IBW | M1108 | 2 | 4515324 | 0.004292871 | 149.61 | 4.84 |
| 19 | SM | M1224 | 2 | 9157583 | 0.006477206 | 1.23 | 3.05 |
| 20 | IBW | M1250 | 2 | 9853370 | 0.007398936 | 110.44 | 4.24 |
| 21 | IBW | M1301 | 2 | 10783075 | 0.007615411 | 69.35 | 3.97 |
| 22 | SM | M1339 | 2 | 11309499 | 0.007959452 | 1.21 | 2.56 |
| 23 | BS | M1434 | 2 | 20835096 | 0.004194877 | 6.32 | 2.84 |
| 24 | SM | M1454 | 2 | 22285395 | 0.00738681 | 1.70 | 3.27 |
| 25 | SM | M1487 | 2 | 24457535 | 0.006518771 | 3.02 | 3.48 |
| 26 | BS | M1544 | 2 | 25790620 | 0.005924379 | 3.08 | 3.01 |
| 27 | IBW | M1554 | 2 | 25884313 | 0.009166481 | 112.09 | 3.89 |
| 28 | IBW | M1589 | 2 | 26224191 | 0.005706758 | 140.75 | 4.42 |
| 29 | BS | M1604 | 2 | 26653873 | 0.009293746 | 2.21 | 2.38 |
| 30 | IBW | M1648 | 2 | 27446861 | 0.000126208 | 87.76 | 7.85 |
| 31 | SM | M1659 | 2 | 27798889 | 0.008179695 | 1.24 | 2.94 |
| 32 | IBW | M1659 | 2 | 27798889 | 3.29E-05 | 91.99 | 8.63 |
| 33 | BS | M1670 | 2 | 28103340 | 0.005211803 | 6.34 | 3.52 |
| 34 | SM | M1753 | 2 | 35844719 | 0.007008376 | 1.18 | 3.10 |
| 35 | SM | M1800 | 2 | 37491093 | 4.86E-08 | 2.39 | 21.87 |
| 36 | IBW | M1801 | 2 | 37734773 | 0.006214811 | 184.73 | 4.45 |
| 37 | SM | M1813 | 3 | 607692 | 0.005157816 | 1.76 | 3.37 |
| 38 | IBW | M1857 | 3 | 4110840 | 0.003064966 | 70.38 | 4.93 |
| 39 | BS | M1908 | 3 | 9193771 | 0.0094062 | 2.11 | 2.39 |
| 40 | SM | M1960 | 3 | 15839988 | 0.00920154 | 3.64 | 3.01 |
| 41 | SM | M1980 | 3 | 18296521 | 0.00969425 | 1.92 | 2.94 |
| 42 | BS | M2093 | 3 | 32818151 | 0.006670146 | 2.57 | 2.39 |
| 43 | SM | M2209 | 3 | 38610554 | 0.008238165 | 3.68 | 3.26 |
| 44 | IBW | M2209 | 3 | 38610554 | 0.003368485 | 199.84 | 5.12 |
| 45 | BS | M2233 | 3 | 39834449 | 0.008007506 | 3.31 | 2.62 |
| 46 | SM | M2242 | 3 | 40103030 | 0.009840973 | 1.36 | 2.89 |
| 47 | SM | M2331 | 4 | 1721901 | 0.007017307 | 1.00 | 2.86 |
| 48 | BS | M2433 | 4 | 5186399 | 0.003656215 | 2.56 | 2.65 |
| 49 | IBW | M2642 | 4 | 18042121 | 0.009585292 | 56.18 | 3.87 |
| 50 | IBW | M2653 | 4 | 19223863 | 0.003665226 | 60.38 | 4.76 |
| 51 | IBW | M2693 | 4 | 21532437 | 0.00943491 | 58.00 | 3.80 |
| 52 | SM | M2713 | 4 | 23479140 | 0.007846361 | 2.64 | 2.92 |
| 53 | BS | M2771 | 4 | 30556062 | 0.009869318 | 2.24 | 2.44 |
| 54 | BS | M2801 | 4 | 31106061 | 0.002419012 | 6.79 | 3.54 |
| 55 | IBW | M2801 | 4 | 31106061 | 0.002004646 | 182.08 | 5.55 |
| 56 | IBW | M2890 | 5 | 2684757 | 0.008291047 | 178.30 | 4.06 |
| 57 | BS | M2904 | 5 | 3476319 | 0.001073556 | 6.66 | 4.35 |
| 58 | SM | M2949 | 5 | 7102952 | 0.002679948 | 1.86 | 3.63 |
| 59 | IBW | M2949 | 5 | 7102952 | 0.000860257 | 100.90 | 5.89 |
| 60 | SM | M2978 | 5 | 9207987 | 0.000348905 | 2.43 | 4.68 |
| 61 | IBW | M2978 | 5 | 9207987 | 0.002310345 | 101.46 | 4.72 |
| 62 | BS | M2985 | 5 | 9349478 | 8.61E-07 | 4.36 | 6.74 |
| 63 | BS | M3011 | 5 | 10452567 | 0.000630231 | 6.16 | 4.64 |
| 64 | SM | M3029 | 5 | 14011220 | 0.004917668 | 2.44 | 3.87 |
| 65 | IBW | M3063 | 5 | 17164694 | 0.007686533 | 58.81 | 4.03 |
| 66 | SM | M3074 | 5 | 17815398 | 0.007210006 | 1.20 | 3.03 |
| 67 | IBW | M3074 | 5 | 17815398 | 0.005317295 | 61.02 | 4.36 |
| 68 | IBW | M3100 | 5 | 19381931 | 0.005879007 | 66.70 | 4.31 |
| 69 | SM | M3119 | 5 | 20420859 | 0.007689443 | 1.26 | 2.95 |
| 70 | SM | M3123 | 5 | 20775169 | 0.004927442 | 1.16 | 3.13 |
| 71 | IBW | M3130 | 5 | 21053226 | 0.005149172 | 63.51 | 4.17 |
| 72 | SM | M3134 | 5 | 21730594 | 0.003883614 | 1.19 | 3.29 |
| 73 | IBW | M3134 | 5 | 21730594 | 0.004657834 | 57.44 | 4.30 |
| 74 | IBW | M3169 | 5 | 25562751 | 0.001820473 | 66.86 | 5.35 |
| 75 | SM | M3314 | 6 | 2644703 | 0.00296404 | 1.81 | 3.70 |
| 76 | SM | M3457 | 6 | 8231298 | 0.007904798 | 1.23 | 2.83 |
| 77 | IBW | M3543 | 6 | 16532259 | 0.001492868 | 165.03 | 5.93 |
| 78 | BS | M3641 | 6 | 23028222 | 0.006562088 | 2.10 | 2.62 |
| 79 | BS | M3871 | 7 | 1215925 | 0.007566823 | 6.65 | 3.03 |
| 80 | SM | M3904 | 7 | 1287983 | 0.009306929 | 1.20 | 2.38 |
| 81 | BS | M3964 | 7 | 3423329 | 7.30E-06 | 4.94 | 8.30 |
| 82 | IBW | M3964 | 7 | 3423329 | 0.008735758 | 86.82 | 3.87 |
| 83 | IBW | M3981 | 7 | 5311054 | 0.002043904 | 208.41 | 5.64 |
| 84 | SM | M3982 | 7 | 5311076 | 0.004367526 | 3.54 | 3.80 |
| 85 | SM | M4010 | 7 | 8310579 | 0.00289647 | 4.23 | 4.25 |
| 86 | IBW | M4010 | 7 | 8310579 | 0.006917851 | 187.86 | 4.51 |
| 87 | SM | M4051 | 7 | 16056368 | 0.003740008 | 1.79 | 3.55 |
| 88 | IBW | M4177 | 7 | 19643842 | 0.008325323 | 66.79 | 3.87 |
| 89 | SM | M4363 | 7 | 26989208 | 0.004246785 | 1.26 | 3.65 |
| 90 | SM | M4397 | 8 | 757118 | 0.007893675 | 1.15 | 3.09 |
| 91 | IBW | M4481 | 8 | 4674600 | 0.000414651 | 84.39 | 6.99 |
| 92 | BS | M4614 | 8 | 10930792 | 0.007441782 | 2.71 | 2.77 |
| 93 | BS | M4679 | 8 | 18692946 | 0.002604986 | 7.40 | 3.32 |
| 94 | BS | M4815 | 8 | 24372727 | 0.003770988 | 2.74 | 3.01 |
| 95 | BS | M4830 | 8 | 27136028 | 0.009523435 | 2.74 | 2.47 |
| 96 | SM | M4941 | 9 | 3408615 | 0.007443629 | 3.38 | 3.13 |
| 97 | IBW | M4941 | 9 | 3408615 | 0.002977689 | 183.46 | 5.04 |
| 98 | IBW | M5057 | 9 | 9495032 | 0.009678609 | 99.61 | 3.81 |
| 99 | SM | M5367 | 10 | 2185198 | 0.000478143 | 1.58 | 5.19 |
| 100 | IBW | M5475 | 10 | 7468552 | 0.008738317 | 51.36 | 3.61 |
| 101 | SM | M5509 | 10 | 9636308 | 0.003601369 | 1.46 | 3.33 |
| 102 | BS | M5587 | 10 | 13279828 | 0.004925126 | 2.36 | 2.87 |
| 103 | IBW | M5599 | 10 | 13661593 | 0.007729885 | 86.35 | 3.94 |
| 104 | BS | M5600 | 10 | 13905276 | 0.005414667 | 6.80 | 2.84 |
| 105 | SM | M5613 | 10 | 15251741 | 0.00630233 | 1.94 | 2.91 |
| 106 | BS | M5640 | 10 | 17146356 | 0.000253084 | 2.67 | 4.32 |
| 107 | IBW | M5652 | 10 | 17806393 | 0.008786084 | 153.66 | 4.20 |
| 108 | BS | M5654 | 10 | 17964746 | 0.008753872 | 1.82 | 2.34 |
| 109 | IBW | M5702 | 11 | 276305 | 0.0025305 | 184.68 | 5.06 |
| 110 | SM | M5704 | 11 | 283469 | 0.00726392 | 1.36 | 3.13 |
| 111 | IBW | M5735 | 11 | 2982852 | 0.002647486 | 72.75 | 4.89 |
| 112 | BS | M5989 | 11 | 14588046 | 0.002899421 | 3.14 | 3.14 |
| 113 | BS | M6229 | 11 | 21028862 | 0.007100398 | 2.75 | 2.72 |
| 114 | SM | M6256 | 11 | 21224745 | 0.003515726 | 1.99 | 3.62 |
| 115 | SM | M6383 | 12 | 1488551 | 0.003228893 | 1.30 | 3.40 |
| 116 | SM | M6445 | 12 | 6435087 | 0.006584709 | 2.34 | 3.14 |
| 117 | SM | M6455 | 12 | 6739875 | 0.007339523 | 1.50 | 2.78 |
| 118 | IBW | M6456 | 12 | 6766280 | 0.006380287 | 177.09 | 4.23 |
| 119 | IBW | M6497 | 12 | 8010637 | 0.006274531 | 67.36 | 4.02 |
| 120 | SM | M6498 | 12 | 8010763 | 0.007277188 | 1.24 | 2.87 |
| 121 | BS | M6498 | 12 | 8010763 | 1.32E-08 | 4.61 | 10.14 |
| 122 | SM | M6514 | 12 | 8341053 | 0.005292874 | 1.76 | 3.01 |
| 123 | IBW | M6521 | 12 | 8545342 | 0.003131555 | 199.77 | 4.93 |
| 124 | BS | M6592 | 12 | 11125856 | 0.008896542 | 3.25 | 2.40 |
| 125 | IBW | M6620 | 12 | 13243638 | 0.003399191 | 197.78 | 4.97 |
| 126 | IBW | M6622 | 12 | 13243729 | 0.002929187 | 191.75 | 5.17 |

**Supplementary Table 4. Significantly associated genome wide epistatic (SNP-SNP) interactions**

| Trait name | SNP 1† | Chr1* | Position 1‡ | SNP 2†† | Chr2⁑ | Position 2‡‡ | LOD epi§§ | PVEepi (%) ¶ | P-value¶¶ |
| --- | --- | --- | --- | --- | --- | --- | --- | --- | --- |
| BS | M432 | 1 | 21146285 | M2600 | 4 | 15768210 | 4.96 | 0.61 | 0.000137 |
| BS | M432 | 1 | 21146285 | M4018 | 7 | 15518197 | 6.49 | 1.97 | 5.18E-06 |
| BS | M922 | 2 | 1205158 | M2299 | 4 | 1239823 | 5.89 | 1.65 | 1.88E-05 |
| BS | M1580 | 2 | 26029298 | M4405 | 8 | 1096852 | 4.19 | 1.22 | 0.000684 |
| BS | M1821 | 3 | 644029 | M5283 | 9 | 20931590 | 7.07 | 2.99 | 1.47E-06 |
| BS | M1919 | 3 | 9342189 | M2007 | 3 | 23352651 | 3.44 | 0.85 | 0.003234 |
| BS | M1964 | 3 | 16184713 | M5423 | 10 | 4130004 | 7.59 | 2.18 | 4.73E-07 |
| BS | M2042 | 3 | 28311069 | M4795 | 8 | 21843997 | 5.74 | 1.91 | 2.58E-05 |
| BS | M2046 | 3 | 28693403 | M5653 | 10 | 17934494 | 6.15 | 2.62 | 1.06E-05 |
| BS | M2138 | 3 | 33841719 | M6502 | 12 | 8051600 | 5.53 | 2.12 | 4.08E-05 |
| BS | M2492 | 4 | 10218508 | M3706 | 6 | 28974980 | 8.46 | 5.05 | 7.07E-08 |
| BS | M3578 | 6 | 17709846 | M6409 | 12 | 3354567 | 8.38 | 2.46 | 8.42E-08 |
| BS | M4111 | 7 | 19064266 | M5510 | 10 | 9636399 | 3.96 | 1.81 | 0.001108 |
| BS | M4864 | 8 | 28230185 | M6007 | 11 | 15177129 | 5.44 | 1.4 | 4.89E-05 |
| BS | M4900 | 8 | 29204032 | M5687 | 10 | 21339589 | 9.54 | 2.97 | 6.58E-09 |
| GN | M430 | 1 | 20987943 | M6243 | 11 | 21140672 | 3.66 | 2.63 | 0.002069 |
| GN | M1243 | 2 | 9793181 | M6804 | 12 | 21707768 | 4.71 | 3.08 | 0.000231 |
| GN | M1360 | 2 | 12893033 | M6286 | 11 | 21653189 | 4.66 | 2.37 | 0.000258 |
| GN | M1723 | 2 | 31910456 | M4235 | 7 | 20758079 | 9.31 | 3.8 | 1.10E-08 |
| GN | M2105 | 3 | 33192466 | M5263 | 9 | 19766113 | 5.16 | 2.64 | 8.99E-05 |
| GN | M2118 | 3 | 33411263 | M5772 | 11 | 4733285 | 6.48 | 1.78 | 5.32E-06 |
| GN | M2147 | 3 | 34115814 | M3643 | 6 | 23098999 | 4.51 | 3.75 | 0.000351 |
| GN | M2609 | 4 | 15770325 | M3659 | 6 | 25314523 | 6.71 | 2.94 | 3.18E-06 |
| GN | M3180 | 5 | 26638327 | M4868 | 8 | 28409207 | 5.79 | 4.18 | 2.34E-05 |
| GN | M3194 | 5 | 29225324 | M6753 | 12 | 18551221 | 3.44 | 2.52 | 0.00327 |
| GN | M4649 | 8 | 14850463 | M4728 | 8 | 20193005 | 3.41 | 1.69 | 0.000389 |
| IBW | M120 | 1 | 3927999 | M4929 | 9 | 122988 | 4.89 | 3.05 | 0.000157 |
| IBW | M555 | 1 | 24900614 | M5007 | 9 | 6859100 | 7.79 | 2.6 | 3.09E-07 |
| IBW | M811 | 1 | 45665630 | M3521 | 6 | 12759508 | 4.76 | 1.72 | 0.000209 |
| IBW | M922 | 2 | 1205158 | M5241 | 9 | 18603965 | 6.29 | 4.04 | 7.93E-06 |
| IBW | M926 | 2 | 1321519 | M4273 | 7 | 23389593 | 5.44 | 1.69 | 4.89E-05 |
| IBW | M1478 | 2 | 24416408 | M5618 | 10 | 15602784 | 3.35 | 2.05 | 0.003929 |
| IBW | M1717 | 2 | 31528693 | M4548 | 8 | 8210462 | 5.4 | 0.79 | 5.39E-05 |
| IBW | M1821 | 3 | 644029 | M4027 | 7 | 15899338 | 5.68 | 1.17 | 2.92E-05 |
| IBW | M2867 | 5 | 1753024 | M4861 | 8 | 28168386 | 3.27 | 1.05 | 0.004609 |
| IBW | M3689 | 6 | 26809636 | M4389 | 8 | 473487 | 3.6 | 1.58 | 0.002332 |
| IBW | M4461 | 8 | 2925918 | M5240 | 9 | 18512384 | 5.61 | 0.77 | 3.40E-05 |
| IBW | M4896 | 8 | 29076382 | M6007 | 11 | 15177129 | 7.91 | 3.07 | 2.37E-07 |
| IBW | M5384 | 10 | 3015931 | M5961 | 11 | 14221043 | 3.5 | 0.8 | 0.002876 |
| IBW | M5485 | 10 | 7955637 | M5613 | 10 | 15251741 | 5.59 | 2.04 | 3.53E-05 |
| IBW | M5876 | 11 | 7899977 | M6396 | 12 | 1871233 | 6.1 | 2.41 | 1.20E-05 |
| PW | M9 | 1 | 436121 | M3507 | 6 | 11708107 | 5.63 | 4.68 | 3.30E-05 |
| PW | M121 | 1 | 3945246 | M5415 | 10 | 3694373 | 5.68 | 6.85 | 2.92E-05 |
| PW | M513 | 1 | 23235924 | M2371 | 4 | 2952390 | 5.87 | 3.08 | 1.96E-05 |
| PW | M1814 | 3 | 614461 | M5213 | 9 | 17834153 | 6.62 | 9.2 | 3.92E-06 |
| PW | M2021 | 3 | 25068219 | M2422 | 4 | 4469079 | 5.62 | 4.01 | 3.32E-05 |
| PW | M3194 | 5 | 29225324 | M4197 | 7 | 19818963 | 4.81 | 5.96 | 0.000186 |
| SM | M89 | 1 | 3117394 | M4883 | 8 | 28720880 | 3.75 | 2.49 | 0.001722 |
| SM | M148 | 1 | 6557456 | M3567 | 6 | 17361201 | 3.97 | 1.36 | 0.001088 |
| SM | M256 | 1 | 12014415 | M5866 | 11 | 7803957 | 4.65 | 1.18 | 0.000264 |
| SM | M265 | 1 | 13450309 | M5506 | 10 | 9386224 | 6.3 | 3.93 | 7.70E-06 |
| SM | M715 | 1 | 37528027 | M3955 | 7 | 3325397 | 4.81 | 0.6 | 0.000187 |
| SM | M852 | 1 | 46223889 | M5201 | 9 | 16861159 | 9.29 | 4.02 | 1.15E-08 |
| SM | M900 | 2 | 392962 | M1599 | 2 | 26452839 | 3.54 | 1.74 | 0.002635 |
| SM | M1238 | 2 | 9481158 | M2160 | 3 | 35132566 | 3.14 | 1.88 | 0.005939 |
| SM | M1271 | 2 | 10656018 | M3870 | 7 | 1215869 | 3.68 | 1.65 | 0.001962 |
| SM | M1454 | 2 | 22285395 | M5372 | 10 | 2243372 | 5.81 | 2.65 | 2.21E-05 |
| SM | M2359 | 4 | 2716390 | M4966 | 9 | 5364603 | 9.09 | 4.14 | 1.80E-08 |
| SM | M2371 | 4 | 2952390 | M3206 | 5 | 29759493 | 7.03 | 1.47 | 1.62E-06 |
| SM | M3174 | 5 | 26229149 | M3234 | 6 | 424695 | 3.75 | 1.48 | 0.00172 |
| SM | M5646 | 10 | 17310698 | M6514 | 12 | 8341053 | 5.82 | 2.76 | 2.17E-05 |
| SM | M5678 | 10 | 20493926 | M6184 | 11 | 20185222 | 4.04 | 1.44 | 0.000946 |

Note: † SNP 1 and ††SNP 2 are the significant SNPs; * Chromosome ID at the first scanning position; ‡Scanning position in bp of the first SNP; ⁑ Chromosome ID at the second scanning position; ‡‡Scanning position in bp of the second SNP; §§ LODepi score caused by epistatic effects; ¶ PVEepi (%): Phenotypic variation explained by epistatic effects; ¶¶ P-Value is the probability value for the significance of interaction.
